# Neurocognitive dynamics and behavioral differences of symmetry and asymmetry processing in working memory: insights from fNIRS

**DOI:** 10.1038/s41598-024-84988-8

**Published:** 2025-02-08

**Authors:** Izabela Maria Sztuka, Simone Kühn

**Affiliations:** 1 Lise Meitner Group for Environmental Neuroscience, Max Planck Institute for Human Development, Lentzeallee 94, 14195 Berlin, Germany; 2 Department of Psychiatry and Psychotherapy, University Medical Center Hamburg-Eppendorf, Hamburg, Germany; 3 Max Planck-UCL Center for Computational Psychiatry and Ageing Research, Berlin, Germany

**Keywords:** Symmetry, Working memory, fNIRS, Neuroaesthetics, Memory, Cognitive neuroscience, Psychology, Human behaviour

## Abstract

Symmetry is a ubiquitous property of the visual world. It facilitates cognitive processing and fosters aesthetic appeal. Despite its importance to aesthetic experience and perceptual prominence, the integration of symmetry in working memory remains underexplored. In our study, participants engaged in a novel working memory task involving both symmetrical and asymmetrical stimuli, while their brain activity was monitored using functional Near Infrared Spectroscopy (fNIRS). The study revealed that symmetry significantly enhances memory performance. Symmetry significantly improves task performance, with symmetrical stimuli leading to higher accuracy and faster recall than asymmetrical ones, especially under high cognitive load. This effect varies with the type of symmetry, with diagonal symmetry being the most effective. Neuroimaging data showed distinct brain activation patterns when participants processed symmetrical stimuli, particularly in the memory-straining condition. Significant differences in brain activity were observed in various brain regions, with lateral occipital, posterior parietal, medial and dorsolateral prefrontal cortices reacting to symmetry with decreased oxygenated hemoglobin (HbO), while in left orbitofrontal (HbO) and right ventrolateral prefrontal cortex (HbO and HbR) hemoglobin concentration increased. Overall, our findings highlight the complex, region-specific brain activation patterns in response to visual symmetry, emphasizing the nuanced role of symmetry in cognitive processing during memory tasks and their potential implication for creative thinking.

**Supplementary Information:**

The online version contains supplementary material available at 10.1038/s41598-024-84988-8.

## Introduction

### Symmetry in visual cognition: bridging aesthetics and cognition

Symmetry is prevalent throughout the human visual environment and provides an ordered, harmonious, and well-proportioned impression to the observer^1^. It aids in the efficient processing and understanding of spatial and structural aspects of our surroundings and has been shown to be processed robustly and automatically, potentially interacting with cognitive processing^2–6^. The common preference for symmetry is not merely for aesthetic satisfaction^7–9^, but also for its cognitive ease, aligning with Gestalt principles of perceptual organization^10–12^. Our study investigated how symmetry, operating on multiple perceptual levels, impacts visual working memory (WM) performance, extending the understanding of symmetry’s role beyond visual processing into cognition.

### Redefining WM interactions with visual perceptual processes

WM, the cognitive system responsible for temporary information storage and manipulation, relies on the intricate interplay among its components^13,14^. Contemporary models accentuate the nuanced roles of the central executive, episodic buffer, visuospatial sketchpad, and other subsystems in managing sensory information, with capacity limitations influenced by both the quantity and control of information^15–19^. Within this framework, we examined how the inherent symmetry in visual stimuli interacts with these components, hypothesizing a facilitative effect on behavioral WM performance and neural activity during WM performance. The neural investigation of visual WM has evolved from a focus on higher-level cognitive regions to an appreciation for the contribution of lower-level visual regions (for review see:^20,21^. Traditionally, studies emphasized high-level brain regions like the prefrontal and parietal cortices in visual WM tasks. Emerging research revealed that content-specific mnemonic information can be decoded from activity patterns across these brain areas^22–25^. However, there is established theory suggesting that the location of information maintenance depends on the nature of the maintained information^26^. In line with this, many studies have demonstrated that not only prefrontal and parietal regions but also sensory cortices are areas from which WM information is decodable^27–29^. These findings broaden our understanding of the neural substrates involved in WM and pose intriguing questions regarding how symmetry influences these neural processes.

### Symmetry’s cognitive footprint in WM

Howe and Jung^30^ found that symmetrical patterns in spatial arrays are remembered better than asymmetrical ones. They attributed this to the fact that symmetrical patterns are perceived as more aesthetically pleasing and thus easier to encode and recall. Early investigations into symmetry’s role in WM suggested that symmetrical configurations might enhance performance, possibly due to the automatic feature binding^6,31–33^. In their dual-task paradigm, Rossi-Arnaud, Pieroni, and Baddeley^33^ hinted at the possibility that symmetry could enhance the binding of visuospatial features, thus improving recall performance in WM tasks. They further investigated this^6^ by scrutinizing the encoding mechanisms of symmetrical configurations in a dual-task setup. Their results revealed superior recall for vertical symmetry compared to other patterns unaffected by concurrent tasks. However, horizontal and diagonal symmetry necessitated active binding processes. Behavioral WM studies showed that symmetry affected WM performance during classic WM paradigms like change-detection^31^ and dual-task^32^ and Corsi block task^33^. However, a comprehensive understanding of how symmetry interacts with memory load remains elusive. Our study addresses this gap, hypothesizing that symmetry facilitates the encoding and maintenance of visual information in WM, potentially through enhanced internal attention mechanisms^17^ affecting WM storage and processing functions. We propose that the improved recall performance for symmetrical stimuli may occur due to symmetry more efficiently encoding the visual information into memory storage. This enhances processing by inhibiting redundant information and focusing attention, thereby supporting the binding of visuospatial features within and between working memory components and affecting neural activity underlying these processes. That could be possible due to *postulated* internally directed attention managed by the central executive to form bound representations^17^. To assess the impact of symmetry on the processes and visuospatial representation within WM, our study manipulates memory load during a delayed matching to sample task. In the experiment, participants underwent 288 trials, each requiring quick and accurate recall of a target’s location after a brief encoding phase of randomly presented symmetrical or asymmetrical stimuli, followed by a recall phase with spatial indication options, all interspersed with variable intertrial breaks. We assume that by aiding in organizing visual and spatial information, symmetry contributes to the maintenance of integrated representation in WM.

### Cortical dynamics of symmetry processing

Research underscores the pivotal role of cortical brain activity during the performance of cognitive tasks^34^, particularly within prefrontal^35,36^ and parietal brain regions^37–39^ as well as in visual areas^26^. To probe the cortical activity associated with symmetry processing in WM tasks, our study employs functional near-infrared spectroscopy (fNIRS) – a non-invasive neuroimaging technique, able to capture cortical activity, which has been shown to be sensitive to cognitive workload changes^40–43^. In fNIRS studies, neural activation is typically indicated by an increase in oxyhemoglobin (HbO) and a decrease in deoxyhemoglobin (HbR). It’s noted that HbO is particularly sensitive in detecting changes brought on by tasks and is more closely correlated with the BOLD response seen in fMRI, as discussed in research by Luke et al.^44^ and Plichta et al.^45^. By comparing cortical response patterns between cognitive loads, the symmetry of stimuli, and response conditions, we aimed to identify differences in neural activity underlying the processing of symmetrical and asymmetrical stimuli in the context of WM.

Our preregistered analysis (https://aspredicted.org/5jj3n.pdf) primarily targeted cortical regions critical to our task, specifically the prefrontal cortex (PFC) and posterior parietal cortex (PPC), where experimental interventions might reveal functional disparities. The dorsolateral PFC (dlPFC), crucial for executive control^46^ managing and retaining information in WM, exhibits structural and functional sensitivity to cognitive load^47^. The ventrolateral PFC (vlPFC) plays a role in the maintenance of object information and inhibitory control helping to suppress irrelevant information^48,49^ with more lateral part suggested to be involved in determining the task-relevance of visual input^50^. Medial PFC (mPFC) aids in sustained attention and internal goal relevance^51^. Furthermore, the orbitofrontal cortex (OFC) is instrumental in fostering goal-oriented actions based on real-time and recalled information^52^ as well as interfacing cognitive with affective information. Our assumption is that symmetry affects the processes in the areas responsible for storage and processing leading to decreased activation.

In the PPC, the intraparietal sulcus (IPS) is linked with handling both spatial and non-spatial data, proving essential for active information retention, particularly in task-relevant data^53^. The superior parietal lobule (SPL) responds to the amount and intricacy of information^54^, while the angular gyrus (AG) is pivotal in information retrieval processes^55^. Insights from these areas guided our exploration of cortical activity dynamics in WM task, particularly under symmetry-induced conditions, assuming PPC would respond with strong differences between symmetrical and asymmetrical conditions.

Building on Christophel et al.‘s^34^ findings, which imply a role for the occipital cortex in maintaining detailed visual representations in WM, we also focused our analysis on this region, specifically the lateral occipital cortex (LOC). LOC is integral to symmetry perception in the visual system, functioning through a combination of bottom-up sensory processing and top-down cognitive influences^20,56^. It efficiently processes symmetrical patterns, essential for object recognition^57^, by integrating features from both halves of a stimulus and utilizing its bilateral field representation to detect symmetry^3,58^. The LOC’s interaction with the prefrontal cortex (PFC) and posterior parietal cortex (PPC) via the dorsal visual stream further highlights^59^ its role in a broader cognitive context. The PFC, responsible for executive functions, modulates the LOC’s symmetry processing with attention and working memory, while the PPC aids in the spatial and attentional aspects, enhancing the spatial representation and attention directed towards symmetrical patterns. This intricate interplay of direct visual processing with higher-level cognitive modulation underscores the LOC’s pivotal role in the visual cognition of symmetry, embedding this perception within an expansive cognitive and attentional framework^3,58^.

We anticipated uncovering distinct neural signatures in the prefrontal, posterior parietal and lateral occipital cortex associated with the processing of symmetrical versus asymmetrical stimuli under different cognitive loads, providing unprecedented insights into these fundamental cognitive operations. By bridging the gap in understanding the interplay between visual symmetry and WM, our study stands to provide insights into the neurocognitive mechanisms of symmetry processing.

## Methods

### Participants

**Fig. 1:**
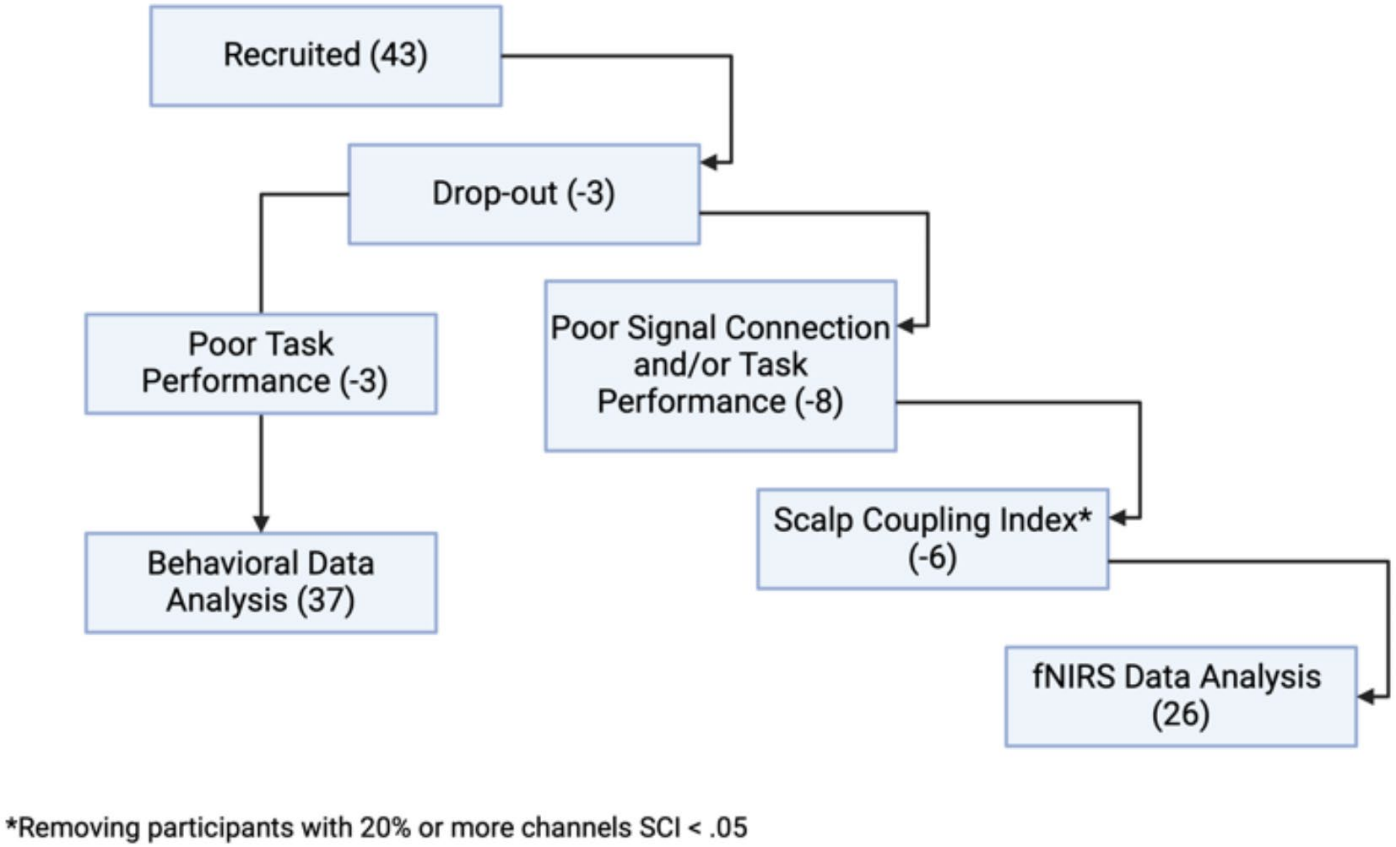
Sample selection procedure.

An a priori power analysis for a repeated measures analysis of variance, conducted using G*Power^60^, indicated that a sample size of 36 participants was needed to achieve 95% power (*α* = 0.05, Cohen’s *f* = 0.25) for detecting a medium effect in a within-subject 2 × 2 design. Consequently, assuming some dropouts from the procedure, we recruited 43 healthy, right-handed subjects aged 18–60 years. All participants provided written, informed consent to participate in the study. Financial compensation was provided to participants. In all procedures, we adhered to the declaration of Helsinki, and the study was approved by the Local Psychological Ethics Board at the University Medical Center Hamburg-Eppendorf (LPEK-0326). All participants reported corrected-to-normal vision. Data exclusion procedure is outlined in Fig. 1. A total of 37 participants were included in behavioral analysis (17 females, age 18–44 years, *M*_*age*_ = 29, *SD*_*ag*e_ = 8), with 3 participants opting to discontinue during the procedure and 3 excluded due to poor task performance. Twenty-six participants were included in the fNIRS analysis (11 females, age 18–44, *M*_*age*_ = 27, *SD*_*ag*e_ = 8). Apart from procedure drop-out, the exclusion criteria also included poor signal connectivity detected post-procedure. We have also excluded participants during data preprocessing, based on signal quality criteria indicating that more than 20% of all channels do not meet good signal criteria (Fig. 1). The post hoc power analysis of the reduced sample for fNIRS analysis revealed a calculated power of approximately 0.85. That means that with 26 participants in our study, we have an 85% chance of correctly rejecting the null hypothesis if there is indeed a true effect of the specified size (effect size f = 0.25). This indicates that despite diminished sample our study remained well-powered for detecting a medium effect size under these conditions.

### Stimuli

Seventy-two novel stimuli were designed for the experiment using MATLAB 2019a software. All stimuli were designed to be meaningless and abstract and aimed to resemble fractal self-similar objects. Such an approach was used by early studies on symmetry perception by Julesz^61^ and Fender and Julesz^62^ .

**Fig. 2:**
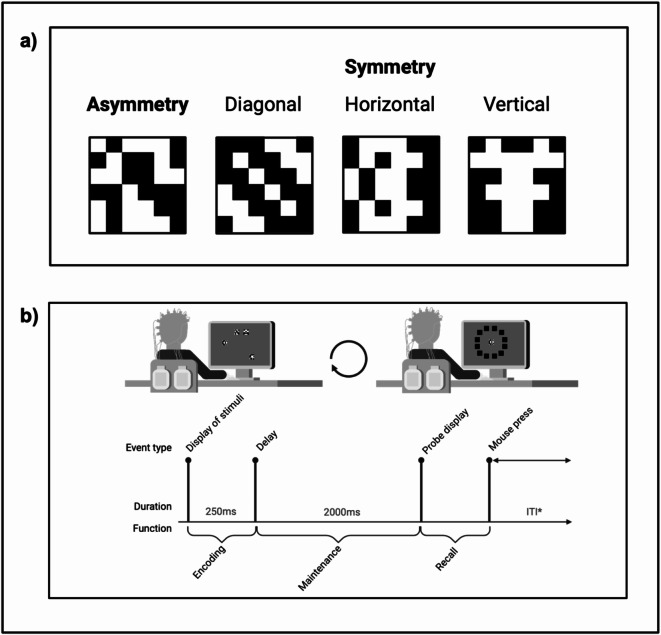
Stimuli and procedure. (**a**) Examples of stimuli used in the paradigm. (**b**) Experimental paradigm and trial schematics with an outline of cognitive stages of the task. * varied ITI timings.

Black-and-white scale was used for optimal visual contrast. For asymmetrical stimuli (*n* = 36), 36 black-and-white square blocks (0 = white; 1 = black) were randomized into six-by-six square 2-dimensional matrices. 50% of the blocks were black. In symmetrical stimuli (*n* = 36) 18 black-and-white square blocks were randomized into three by six rectangular matrices and reflected across horizontal, vertical and diagonal axes. 12 stimuli were designed in each of the three main types of symmetry: vertical, horizontal and diagonal (Fig. 2a).

After generating the matrices, the stimulus images were saved in TIFF format. To quantify the amount of information carried in each image, we employed a compression-based method previously utilized in studies on visual complexity (e.g.^63–65^). Specifically, the stimuli were converted to JPEG format, and the resulting file sizes provided an indirect but effective measure of image complexity, as JPEG compression reduces file size by discarding redundant or less critical information. We assumed that asymmetrical images as more complex retain more data even after compression, leading to larger file sizes, whereas symmetrical as simpler images compress into smaller files.

Research supports the use of compression-based measures as valid proxies for visual complexity, with such methods correlating positively with subjective complexity judgments^65^. Additionally, Machado et al.^64^ highlight that compression size provides an objective computational metric to quantify complexity, offering a reliable estimate of the informational content in visual stimuli. Given these established findings, the JPEG compression sizes of symmetrical and asymmetrical images were statistically compared using independent samples t-test indicating a signiticant difference (*t*(81.91) = -4.19, *p* < 0.001) indicating that compression for asymmetrical stimuli was higher than for symmetrical stimuli (*M* = 1228.0, *SD* = 75.7 for asymmetrical; *M* = 1148.0, *SD* = 98.0 for symmetrical). Therefore, we chose to incorporate compression rate as random effects in the supplementary behavioral analysis to account for stimulus complexity and its potential influence on perceptual and cognitive performance.

### Design

A full-factorial, repeated measures design was used in this experiment. Independent variables were: symmetry (with two levels: symmetrical and asymmetrical) and load (number of displayed items: two or four). Dependent variables were measured as the correct answer (accuracy) and response time. For additional testing of the hypothesis that there are differences in accuracy of recall between different levels of symmetry, we assumed that the independent variable type has 3 levels of symmetry (vertical, horizontal, diagonal). To test the hypothesis about the impact of symmetry on error responses the dependent variable was distance of error location from target and the angle of location and the number of false-positive responses. The experiment consisted of four blocks of 72 trials with a 30-second break between blocks.

### Procedure

The experiment was designed and programmed using MATLAB 2019a software and the Psychtoolbox (ver 3.0.15)^66^. Testing took place in a quiet and dark room, with participants seated 60 cm from the monitor. Initially, participants filled out an informed consent form and a demographic questionnaire. They were then connected to electrocardiogram (ECG) electrodes for physiological measurement, and an fNIRS cap was fitted to their heads. Calibration was performed to ensure signal quality before recording began.

Participants were instructed to close their eyes for a baseline measurement until they heard a loud sound after 5 min. Subsequently, instructions for the main tasks were displayed on the screen. Participants read the instructions and proceeded when ready; no practice trials were included due to the task’s straightforward nature.

The main objective for participants was to accurately and swiftly recall the location of the target probe. The experiment comprised four blocks of 72 trials each, totaling 288 trials. Each trial included encoding and recall phases, separated by a maintenance phase (Fig. 2b). Trials began with an intertrial interval (ITI) displaying a cross for 2, 4, 6, 8, or 10 s^67,68^. During the encoding phase, two or four stimuli (cognitive load condition: low and high) were randomly presented at one of 12 locations on the screen for 250ms. These locations were computed as vertices of a dodecagon with a 200-pixel radius, centered on the screen. Each vertex marked the center (anchor) of one of 12 square locations (75 pixels each), where visual stimuli were displayed. Each set included one target stimulus and either one or three distractors, with 50% of the stimuli being symmetrical, following paradigms in cognitive neuroscience research on visual working memory^69,70^.

Following the encoding phase, a 2000ms maintenance phase ensued, without a fixation point displayed. (as shown in Fig. 2b). In the recall phase, a target probe matching one of the stimuli from the encoding phase was presented at the center of the screen, with 12 black squares indicating the possible locations of the target probe. Participants had a 10-second response window to decide the location. After each recall phase, the mouse cursor was reset to the center of the screen for the next trial. All 72 stimuli used in the experiment were presented in random order. The complete task took an average of 42 min to complete.

### fNIRS acquisition

#### fNIRS device and acquisition parameters

Recording was obtained using two NIRSport2 (NIRx Medizintechnik GmbH, Germany) optical imaging systems (16 × 16) resulting in a 32 × 32 setup. The NIRSport2 used dual LED source illumination and Avalanche Photodiode (APD) type detectors operating at wavelengths of 760 nm and 850 nm. Data were collected at a sampling rate of 5.08 Hz from 116 channels. The neurophysiological data were additionally supplemented with simultaneous physiological recording via the NIRxWings module of ECG, oxygen saturation (SpO2) and photoplethysmography (PPG). The purpose of the recording was to aid filtering of systemic physiological noise occurring during the experiment. Functional and physiological data were acquired using Aurora acquisition software (v2021.9.0.12, NIRx Medizintechnik GmbH, Germany).

#### Optode array design

**Fig. 3:**
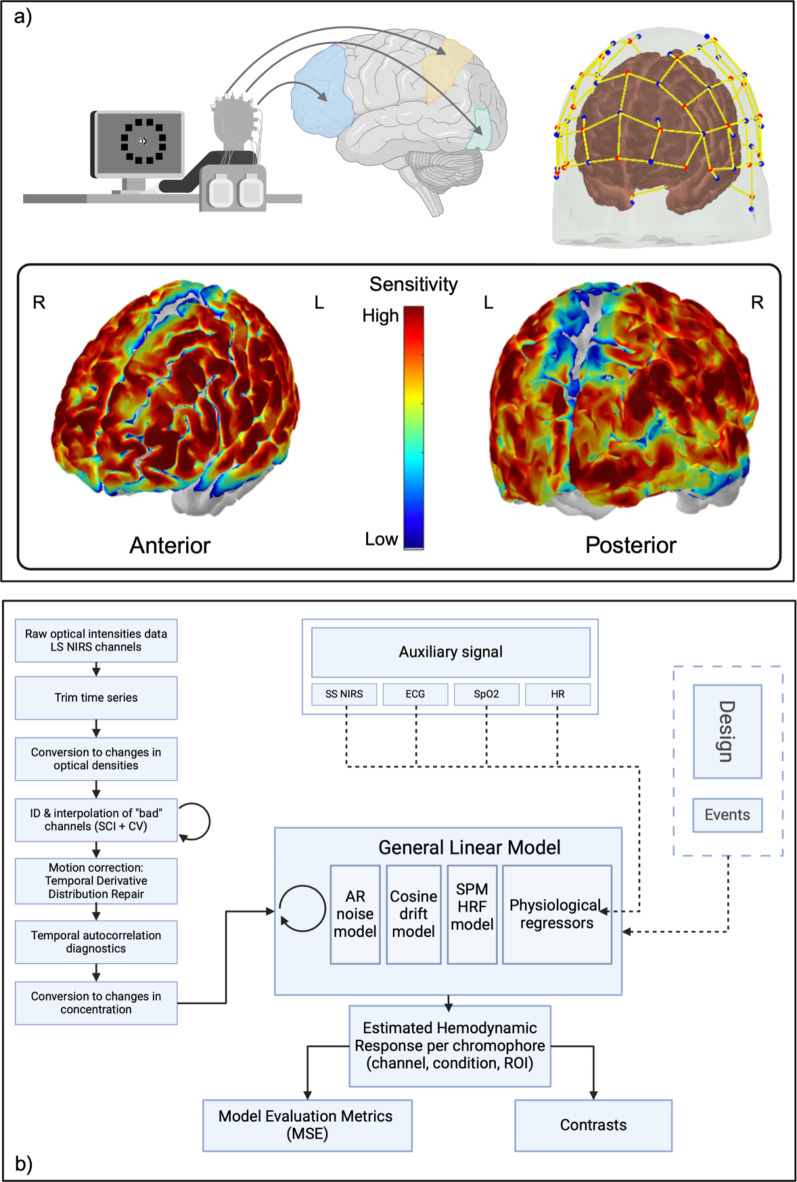
Neurophysiological measurement: (**a**) Hypothesized effect of task on key regions of interest (top left); fNIRS: optode layout with channel array, blue – detectors, red – sources, short channels with short yellow lines (top right); Posterior and anterior hemisphere cortical sensitivity profile of the montage, generated using Monte Carlo simulation in AtlasViewer toolbox. The sensitivity values are displayed in log10 units. (**b**) Schematic representation of fNIRS preprocessing steps and subject-level analysis (SS – Short separation channels; ECG – electrocardiogram; SpO2 – oxygen saturation; HR – heart rate; AR – autoregressive; SPM – statistical parameter modelling; HRF – hemodynamic response function).

To obtain fNIRS signal we have designed a whole-brain optode array using 64 optodes (32 sources and 32 detectors). Optode coordinates were determined based on the EEG 10–20 reference system (Fig. 3a). The optode positioning was generated using NIRSite software package (version 2.0, NIRx Medizintechnik GmbH, Germany) with coordinates identified in MNI space. Moreover, as recommended in the literature^71,72^, 16 8 mm short-separation detectors were added to the montage. The signal from these channels was used during the noise reduction procedure of extracerebral confounds in the General Linear Model^73,74^ to help reduce false discovery rates^75^. The layout provided recording from 116 channels with a long channel intra-optode distance of 30 mm and 8 mm for short channels. The spatial registration procedure to estimate cortical regions covered by fNIRS channels and their sensitivity profile (Fig. 3a, bottom) was performed using Monte Carlo simulation in AtlasViewer^76^. The placement of fNIRS optodes on participants was standardized with reference to the nasion and eyebrows. These landmarks functioned as intermediaries for transforming real-world coordinates into the Montreal Neurological Institute (MNI) coordinates. This conversion, crucial for aligning with a standard MRI brain atlas, was accomplished by applying an affine transformation. This approach ensured consistent positioning across participants, allowing for minor positional variations within a range of approximately 5 mm. Full documentation to reproduce the optode setup used in the present experiment is available via OSF repository^77^.

#### Regions of interest (ROI)

In our study, we focused on key brain regions involved in working memory (WM) and symmetry processes. Our ROIs included the Prefrontal Cortex (PFC), which bilaterally encompasses 32 channels and functionally covered dlPFC, OFC, mPFC, and vlPFC (broadly Brodmann areas 9, 10, 44, 45, and 46), the Posterior Parietal Cortex (PPC) with 15 channels, and the Lateral Occipital Cortex (LOC) consisting of 13 channels. Additionally, as controls, we analyzed channels over the left/right motor area (18 channels), sensitive to motor responses associated with location selection, and the early visual area activated by visual stimuli - comprising 8 channels. It is important to note that while WM content can be decoded from early visual areas^28,29^, univariate analysis in WM studies has been criticized for potentially reflecting not just WM processing but also supporting functions such as attention. Given that the study controls for load rather than attention our results from early visual areas may reflect combination of factors rather than solely WM processes. Detailed information on the segmentation of channels into these ROIs is provided in Supplementary Material: ROI, offering insights into our methodology for isolating specific brain activities within each region.

### Data analysis

#### Behavioural

The behavioral data analysis was conducted in R (ver. 4.3.2). It involved several steps to ensure accuracy and provide meaningful results. Initially, the data from the experiment were processed, with total accuracies and means calculated for each participant and condition. To ensure the reliability of the results, an initial inspection of the reaction time data distribution was conducted. To identify potential inaccuracies, standard diagnostic procedures were applied to scrutinize the data and detect abnormalities in the predicted distribution. Outliers, defined as reaction times outside of the margin of double standard deviation from the mean^78^, were subsequently removed from the dataset.

Additional derivatives extracted during the experiment were also preprocessed. The angle of error response relative to the center-target location baseline (computed using the inverse cosine function (*arccos*) provided insights into the direction and magnitude of errors. Furthermore, distance from the target and false-positive response rate (participant selecting a distractor location, a rate calculated as proportion of all incorrect responses) were calculated. These variables played a crucial role in the supplementary analysis, specifically focusing on the location of errors. By following this approach, the analysis ensured a comprehensive evaluation of participants’ performances, identification of potential outliers and deeper insights into the nature and patterns of errors.

As a final step, in order to relate the behavioral outcome to neural activities evoked by the task, we have computed for each participant two derivatives of accuracy performance in the task. The first covariate accounted for the difference between the performance under symmetrical vs. asymmetrical probe (accuracy ^sub^_symmetry_ – accuracy ^sub^_asymmetry_). The second, considered the difference in performance between the cognitive load conditions (accuracy ^sub^_low_ – accuracy ^sub^_high_). These metrics were used during fNIRS analysis as a proxy for differences in neural activations and behavioral performance.

To answer the hypothesis of an effect of symmetry on WM, behavioral data were analyzed with repeated measures analysis of variance (ANOVA). The main model’s predictors included effect of probe type and cognitive load as well as their interactive term. Separately, they predicted accuracy in the task and reaction time to the probe in correct trials. Moreover, we examined the relationship between stimulus complexity, quantified via JPEG compression rate, and task performance measures, including accuracy and reaction time. To this end, Pearson’s correlations were computed between JPEG compression rates and both accuracy and RT across trials. Auxiliary models predicted the distance of error from the target, false positives as well as accuracy in response to probe type and cognitive load. The analysis was conducted using R’s *ez p*ackage (ver 4.4.0)^79^. Degrees of freedom were corrected if non-sphericity was detected (Greenhouse-Geisser (GG) correction). Moreover, we conducted posthoc pairwise tests with Bonferroni correction using the *emmeans* package in R (ver 1.8.9)^80^.

#### fNIRS

Following the collection of data, all the gathered information was transformed into the Brain Imaging Data Structure (BIDS) format, as outlined by Gorgolewski et al.^81^. This conversion was accomplished using the MNE-BIDS tool^82^. The signal preprocessing and analysis of event-related responses were performed using *mne*^83^ and *mne-nirs*^44^. Statistical analysis was executed with Python’s *statsmodels*^84^, R’s *lme4*^85^ and *eemeans* package. The code to reproduce the analysis is available via the open access repository^77^. fNIRS data analysis involved the following steps:

##### Preprocessing

First, we preprocessed functional data, which involved several key steps (Fig. 3b). Initially, raw intensity was converted to optical density (OD) signals. Subsequently, to assess signal quality metrics, we extracted the scalp coupling index (SCI)^86^ and calculated coefficient of variation (CV) to measure the connection quality between the optode and scalp following procedure outlined by Piper et al.^87^. Using both measures, all channels with SCI < 0.05 and CV > 2 standard deviations from the mean of the signal were excluded from further analysis. For each participant, the signal was also scrutinized for motion artefacts with temporal derivative distribution repair^88^ to correct baseline shifts and spike artefacts. Following this step, we have converted ODs to hemoglobin concentrations using Beer-Lambert law with a partial pathlength factor equal to 1. Preprocessing also included functions to extract condition-related data, such as adjustment of fixed post-onset event duration to 250ms to ensure the analysis was consistent across all data.

##### Subject-level model

Estimating changes in hemodynamic response (for HbO and HbR) was conducted first at an individual subject level. To mitigate the impact of confounding systemic signals, as a final step, we have added data from short channels and ECG as regressors to the GLM. Incorporating the ECG signal directly into the GLM as a regressor allows for the correction of systemic physiological fluctuations related to cardiac cycles. We have chosen not to apply any spatial frequency filters due to the steps applied during subject-level GLM analysis.

We have used a GLM with an autoregressive noise model (AR^1^-GLM) for this analysis. The AR-GLM allows us to account for temporal autocorrelation structure in the fNIRS time series. Such an approach is validated and well-established within NIRS research on cognitive processes^67,72^. Our analysis also included a canonical model of SPM (*nilearn ‘spm’ function*) in GLM as the hemodynamic response with time and dispersion derivative regressors. The cosine basis function accounted for signal drifts over time. Additionally, a high-pass filter with a cut-off frequency of 0.03 Hz is applied to remove low-frequency noise. The cut-off frequency for the high-pass filter was determined considering the longest time period between the events. Short-channel regressors for HbO and HbR signals were added to the design matrix^89,90^. These regressors account for systemic physiological changes measured by short separation (SS) channels. Auxiliary biophysical data (ECG, SpO2) were also added to the design matrix^91^ (Fig. 3b), in order to account for physiological variations that might influence the fNIRS signals.

Resulting GLM estimates were used to ascertain the changes in chromophore (HbO and HbR) concentration within our ROI and to estimate the activity contrast of our hypothesized effect of symmetry on neural activity.

##### Statistical analysis

To obtain group-level results, we have statistically evaluated channel-wise contrasts and ROI-level activity with linear mixed-effects (LME) models. In our models, we have evaluated how the effect size of chroma varies with the effect of probe type and encoded load while also accounting for the random effect of the individual measurement. To this end, the model includes both the main effects and the interaction between probe type and encoded load. Models were performed separately per contrast, ROI, and chroma.

In functional near-infrared spectroscopy (fNIRS) studies, the coefficients from GLM typically represent the estimated change in the concentration of hemoglobin (HbO or HbR) in response to the experimental conditions or contrasts being tested. These changes are usually expressed in micromolar units (µM), which quantify the magnitude of the hemodynamic response associated with neural activity in the brain. Thus, in our study, if the coefficients in the results are presented without explicit units, they are indicating the change in hemoglobin concentration in micromolar units (µM).

## Results

### Effect of symmetry on accuracy

**Fig. 4:**
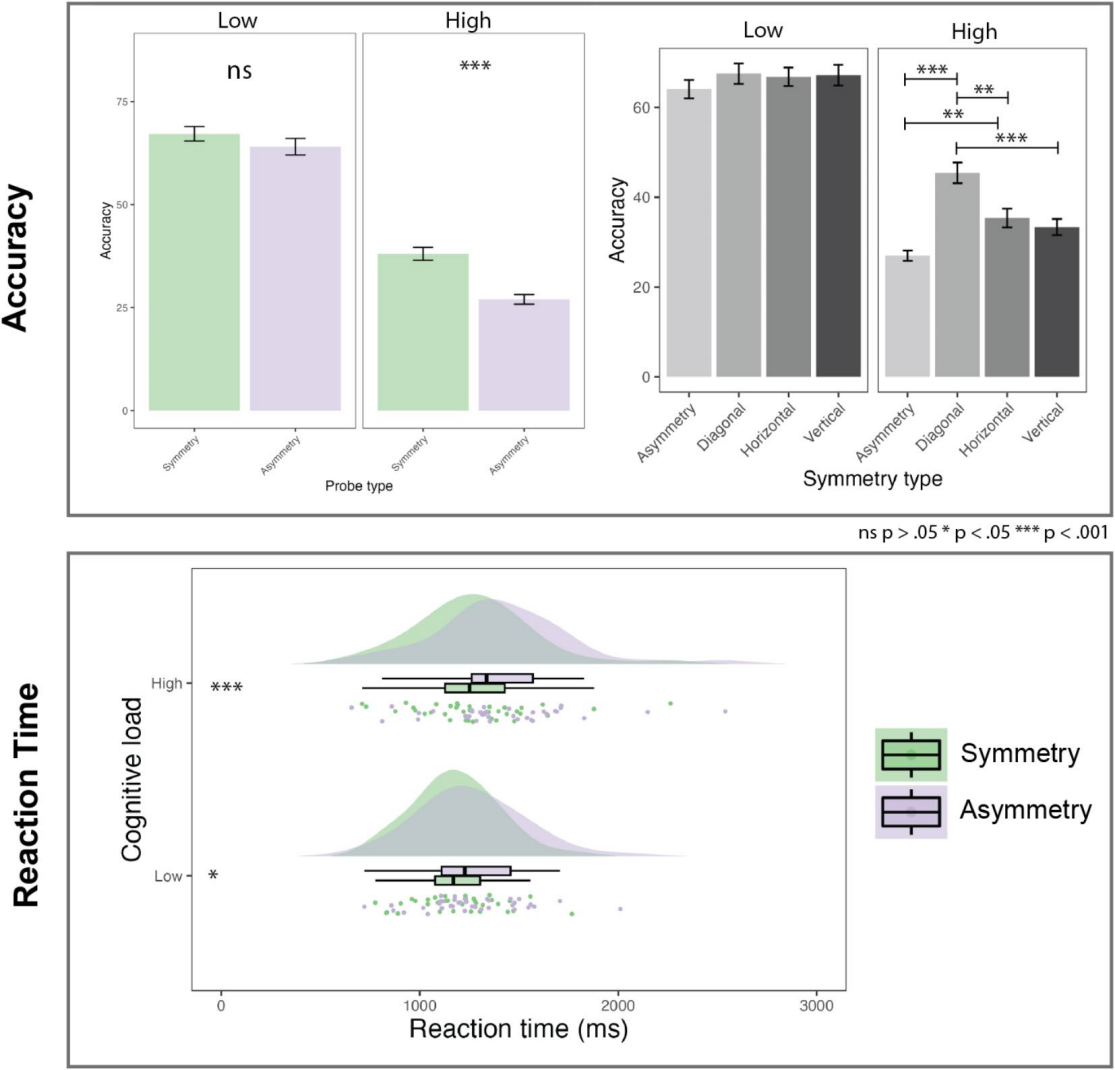
Supplementary results of behavioral analysis: error of distance from the target; angle of error from the target in respect to displayed probe; false positive rate of incorrect responses. All plots have denoted significant pairwise comparisons.

**Fig. 5:**
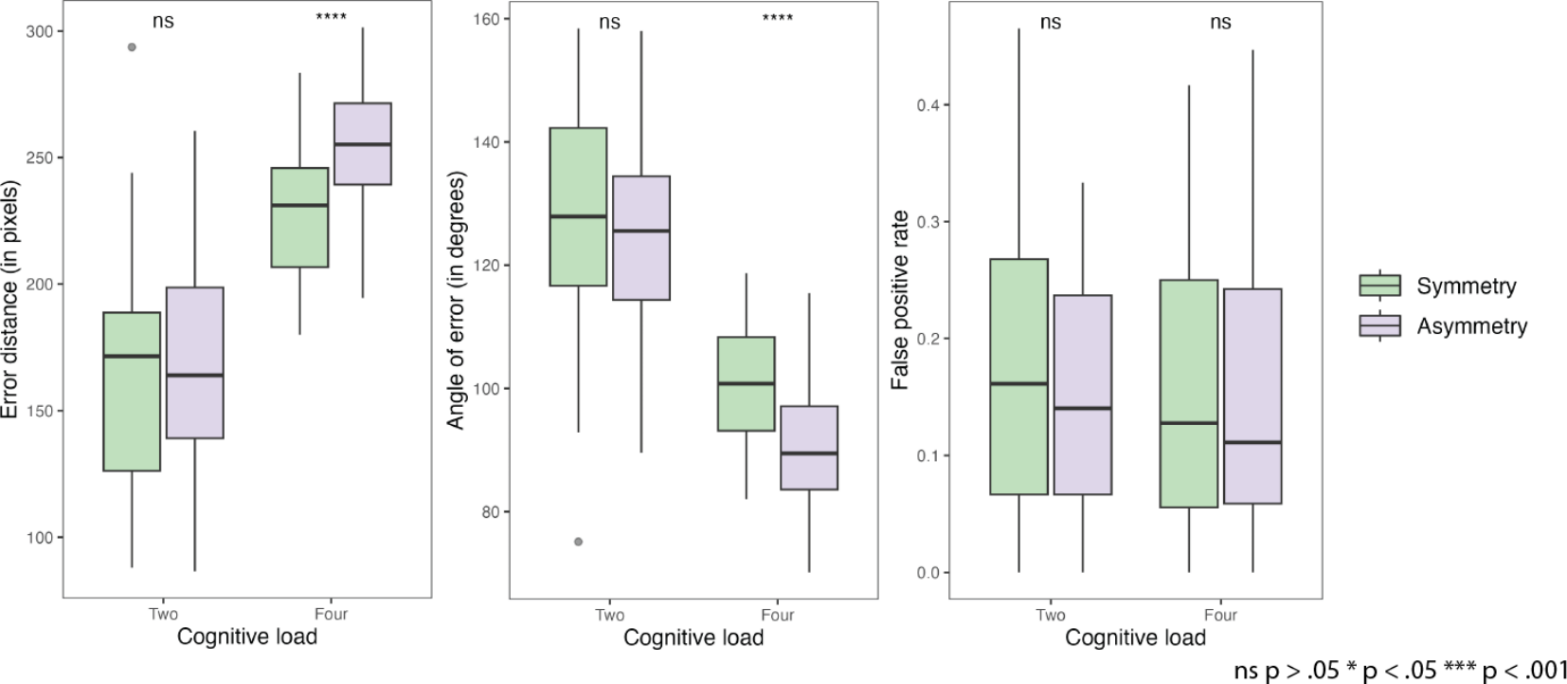
Main results of behavioral analysis: accuracy (in %) by condition with pairwise comparison significance, accuracy by symmetry type (the significance of comparisons available in Supplementary Materials: Extended Data), and reaction time for correct trials by condition with pairwise comparison significance.

The data revealed significant differences in recall accuracy between symmetrical and asymmetrical stimuli (Fig. 4). In general, the accuracy in recall of low cognitive load (2 items) (*M* = 65.61, *SD* = 11.55), was higher than high (4 items) (*M* = 32.51, *SD* = 10.05). The accuracy of recalling symmetrical probe was higher (*M* = 52.61, *SD* = 17.80) than asymmetrical probe (*M* = 45.52, *SD* = 21.16). The two-way repeated measures ANOVA (Supplementary Materials: Table 1) indicated a significant effect of probe type (*F*^1,36^ = 63.29, *p* < 0.001, *η*^2^*G *= 0.112) on accuracy. The task effect was dominated by the cognitive load (*F*^1,36^ = 845.16, *p* < 0.001, *η*^2^*G *= 0.734). The interactive effect between the symmetry and load was also significant (*F*^1,36^ = 16.02, *p* < 0.001, *η*^2^*G* = 0.038), suggesting that differences between symmetry and asymmetry significantly increase with the number of encoded stimuli (see Fig. 5).

In the analysis of pairwise comparisons, significant differences were found for certain contrasts. For low cognitive load, comparing symmetrical probe with asymmetrical probe, the difference was not statistically significant, with an estimated *M*_difference_ = 3.11 (*SE* = 1.43, *t*(108) = 2.17, *p* = .190). This suggests that the difference in accuracy between the symmetrical and asymmetrical probe was minimal. In contrast, comparing symmetry with asymmetry under high cognitive load, there was a significant increase in accuracy, with an estimated *M*_difference_ = 11.05 (*SE* = 1.43, *t*(108) = 7.72, *p* < 0.001). This indicates that a symmetrical probe had a higher accuracy than an asymmetrical probe by approximately 11%.

Finally, we found a weak significant negative correlation between stimulus complexity (as quantified by JPEG compression rate) and task accuracy (*r* = -0.184, 95% *CI* [-0.34, -0.02], *t*(146) = -2.26, *p* = 0.025). This indicates that increased visual complexity was associated with decreased accuracy.

### Effect of symmetry type on accuracy

Expanding on the main results, we have investigated the effect of the type of symmetry (vertical, horizontal, diagonal, asymmetry) on accuracy. Summary statistics are available in Supplementary Material: Table 2. In the analysis of variance (Supplementary Material: Table 3), a significant main effect was observed for symmetry type (*F*(2.50, 90) = 17.78, *p* < 0.001, *η*^2^*G* = 0.09), after adjusting for violations of sphericity with an *ε* value of 0.83, reflecting a moderate effect size.

Additionally, the main effect of the encoded load was significant (*F*^1,36^ = 827.07, *p* < 0.001, *η*^2^*G* = 0.62), denoting a substantial effect of the load on accuracy. Furthermore, the interaction effect between symmetry type and cognitive load was significant (*F*(2.80, 100.85) = 9.41, *p* < 0.001, *η*^2^*G* = 0.05), with an *ε* of 0.93, indicating a small but reliable interaction effect size. These results suggest that both encoded load and symmetry type are significant predictors of accuracy and interact with each other. The high epsilon values suggest that the assumption of sphericity was reasonably met for these effects.

In a post-hoc analysis of pairwise differences, we identified several significant differences in accuracies between symmetry types (Fig. 5). There was a significant difference in high cognitive load between asymmetrical probe and diagonal, with an estimated *M*_difference_ = -18.43 (*SE* = 2.11, *t*(252) = -8.69, *p* < 0.001) and between asymmetrical and horizontal with *M*_difference_ = − 8.39 (*SE* = 2.11, t(252) = -3.95, *p* = 0.003). Furthermore, also in high cognitive load diagonal probe had significantly higher accuracy than horizontal with *M*_difference_ = 8.91 (*SE* = 2.14, *t*(238) = 4.16, *p* = 0.001). Another significant increase was observed for diagonal probe compared to vertical one, with an estimated *M*_difference_ = 10.04 (*SE* = 2.11, *t*(252) = 4.73, *p* < 0.001). Outcomes for all comparisons are available in Supplementary Materials: Extended Tables.

### Effect of symmetry on reaction time

We have focused our investigation of reaction times on differences in correct recall of symmetry and asymmetry (Fig. 5). Descriptive statistics indicated that symmetry had a faster recall response time (Table 1).

**Table 1:** Reaction time by condition (in ms).

Cognitive load	Probe type	M	SD	Max.	Min.
High	Asymmetry	1409.002	540.977	3626.288	499.279
High	Symmetry	1283.717	535.512	3563.625	436.079
Low	Asymmetry	1246.709	477.828	3592.715	444.030
Low	Symmetry	1183.439	440.243	3608.561	466.127

A two-way ANOVA found (Supplementary Material: Table 4) that main effect of probe type was significant, (*F*^1,36^ = 57.11, *p* < 0.001, *η*^2^*G* = 0.033), indicating a small to moderate effect size. The main effect of cognitive load was also significant, *F*^1,36^ = 26.48, *p* < 0.001, *η*^2^*G* = 0.046, suggesting a small to moderate effect size. Additionally, there was a significant interaction effect between probe type and cognitive load, (*F*^1,36^ = 5.15, *p* = 0.029, *η*^2^*G* = 0.003), indicating a small effect size. These results suggest significant effects for both probe type and cognitive load as well as their interaction on reaction times, even after adjusting for sphericity violations.

The post-hoc analysis of pairwise comparisons revealed the difference in low cognitive load condition between asymmetry and symmetry to be significant with *M*_difference_ = 74.24 (*SE* = 24.63, *t*(108) = 3.01, *p* = 0.019). In contrast, the comparison between probe types in high cognitive load revealed a statistically significant *M*_difference_ = 131.06 (*SE* = 24.63, *t*(108) = 5.32, *p* < 0.001). This significant result suggests a robust difference in the means of reaction times with the mean for asymmetrical probe being substantially higher.

A statistically significant but very weak positive correlation was observed between stimulus complexity and reaction time (*r* = 0.041, 95% *CI* [0.022, 0.060], *p* < 0.001). Although significant, the effect size is minimal, suggesting that increased visual complexity had a negligible impact on reaction time.

### Effect of symmetry on errors

In the subsequent analysis, we focused on patterns of erroneous answers (Fig. 5). A two-way ANOVA (Supplementary Material: Table 5) was conducted to assess the impact of probe type and cognitive load on the distance of error from the target. The main effect of probe type was significant, (*F*^1,36^ = 12.48, *p* = 0.001, *η*^2^*G* = 0.053), indicating a moderate effect size. This suggests that the type of probe used had a reliable impact on the error distance. Similarly, the main effect of cognitive load was found to be significant, (*F*^1,36^ = 212.91, *p* < 0.001, *η*^2^*G* = 0.522), denoting a large effect size. This demonstrates that the memory load significantly influenced the error distance. The interaction between probe type and cognitive load was significant, (*F*^1,36^ = 5.25, *p* = 0.028, *η*^2^*G* = 0.018), just below the conventional 0.05 threshold for significance. This suggests that the combined effect of probe type and display condition on error distance was marginally different from the effect of either factor alone.

However, a closer look at pairwise comparisons revealed that the differences between probe types in error of distance are primarily impacted only by high cognitive load with *M*_difference_ = − 26.44 (*SE* = 6.65, *t*(108) = -3.96, *p* = 0.001), suggesting that asymmetry is significantly further away from the target than symmetry. In low cognitive load, that difference is not significant (*M*_difference_ = -7.17, *SE* = 6.65, *t*(108) = -1.07, *p* = 1). We can assume both probe type and cognitive load are important factors affecting the spatial accuracy of responses, as measured by the distance of error from the target.

A repeated measures ANOVA was conducted to assess the influence of experimental conditions on the angle of error of the response (Supplementary Material: Table 6). The analysis revealed a significant main effect of probe type, *F*^1,36^ = 14.11, *p* = 0.001, *η*^2^*G* = 0.062, suggesting a moderate effect size. This indicates that the type of probe used had a reliable impact on the angle of error.

Similarly, the main effect of cognitive load was found to be significant, (*F*^1,36^ = 209.13, *p* < 0.001, *η*^2^*G* = 0.523), denoting a large effect size. This demonstrates that the cognitive load condition significantly influenced the angle of error. However, the interaction effect between probe type and cognitive load was marginally not statistically significant, *F*^1,36^ = 3.18, *p* = 0.059, *η*^2^*G* = 0.015, suggesting that the combined effect of probe type and display condition on the angle of error was not significantly different from the effect of either factor alone.

These findings indicate that both probe type and cognitive load are important factors affecting the angle of error, with significant main effects observed for each. The lack of a significant interaction effect implies that these factors do not interact to further influence the angle of error in the conditions tested.

The post-hoc pairwise comparisons revealed that the angle of error differences between asymmetrical and symmetrical probes were only significant in the high cognitive load conditions (*M*_difference_ = 10.94 (*SE* = 2.77, *t*(108) = 2.94, *p* = 0.001).

Furthermore, to detect if a symmetrical probe was making it more likely for participants to select another displayed location, we analysed false positives (participants selecting other previously displayed locations). For each participant, the false positive rate was calculated as the proportion of false positive answers to all incorrect answers. A two-way ANOVA showed (Supplementary Material: Table 7) that there was no significant effect of symmetry (*p* = 0.488) or cognitive load (*p* = .916) and their interaction (*p* = 0.444) on false positive rate. Multiple comparisons test indicated no significant differences between the conditions (*p* = 1).

### Effect of symmetry on regional cortical activations

Our study premise considered that the symmetrical probe will have an effect on activity within WM-related brain regions, notably PFC and PPC. Moreover, we hypothesized that LOC, which is heavily affected by symmetry perception, could also show differences in the processing of symmetry and asymmetry during the memory task. We have tested our hypothesis using linear mixed modeling to infer a group-level conclusion on hemoglobin concentration changes in ROIs in response to our conditions. In the case of PFC, we chose to test the differences within distinct cortical components of PFC: dlPFC, OFC, mPFC and vlPFC. The PFC components, PPC and LOC were analyzed and are reported hemisphere-wise. Such an approach aimed to provide a fine-grained picture of cortical dynamics during the task. The outcomes for particular ROI are presented below.

*Prefrontal Cortex*: We have conducted a detailed analysis of distinct cortical components of PFC. For clarity, this manuscript reports instances where the model was statistically significant for both HbO and HbR or with behavioral covariate. Detailed results including model metrics and performance, are available in Supplementary Material: Neuroimaging results. The LME indicated a significant effect of the symmetrical probe on an increase of HbO and decrease of HbR in right dlPFC (*β*_*HbO*_ = 7.03, *SE*_*HbO*_ = 1.50, *p*_*HbO*_ < 0.001; *β*_*HbR*_ = -1.61, *SE*_*HbR*_ = 0.48, *p*_*HbR*_ = 0.001) as well as significant interaction between symmetrical probe and cognitive load with decrease in HbO (*β*_*HbO*_ = -18.24, *SE*_*HbO*_ = 2.12, *p*_*HbO*_ < 0.001) and increase in HbR (*β*_*HbR*_ = 3.45, *SE*_*HbR*_ = 0.67, *p*_*HbR*_ < 0.001). Furthermore, left OFC indicated being sensitive to significant interaction between probe type and cognitive load (*β*_*HbO*_ = -9.53, *SE*_*HbO*_ = 2.61, *p*_*HbO*_ < 0.001; *β*_*HbR*_ = 2.48, *SE*_*HbR*_ = 0.91, *p*_*HbR*_ = 0.007). Moreover, results showed a significant effect of accuracy difference between symmetry and asymmetry in HbO (*β*_*HbO*_ = 324.19, *SE*_*HbO*_ = 156.51, *p*_*HbO*_ = 0.039) but not for HbR (*p* = 0.600).

In mPFC the LME indicated a significant effect of symmetrical probe type on HbO (*β*_*HbO*_ = 9.29, *SE*_*HbO*_ = 1.25, *p*_*HbO*_ < 0.001), the HbR was affected in a marginally insignificant way (*β*_*HbR*_ = -0.85, *SE*_*HbR*_ = 0.45, *p*_*HbR*_ = 0.057). Interestingly, HbO and HbR in right vlPFC were simultaneously significantly decreased by symmetrical probe type (*β*_*HbO*_ = -6.20, *SE*_*HbO*_ = 2.67, *p*_*HbO*_ = 0.020; *β*_*HbR*_ = -4.29, *SE*_*HbR*_ = 1.19, *p*_*HbR*_ < 0.001) while in left vlPFC there was a significant interaction between probe type and cognitive load increasing both HbO and HbR (*β*_*HbO*_ = 22.47, *SE*_*HbO*_ = 2.59, *p*_*HbO*_ < 0.001; *β*_*HbR*_ = 4.37, *SE*_*HbR*_ = 1.11, *p*_*HbR*_ < 0.001). In all models, we observed distinct random effects structures for HbO and HbR with the HbO model exhibiting higher variability than HbR.

**Fig. 6:**
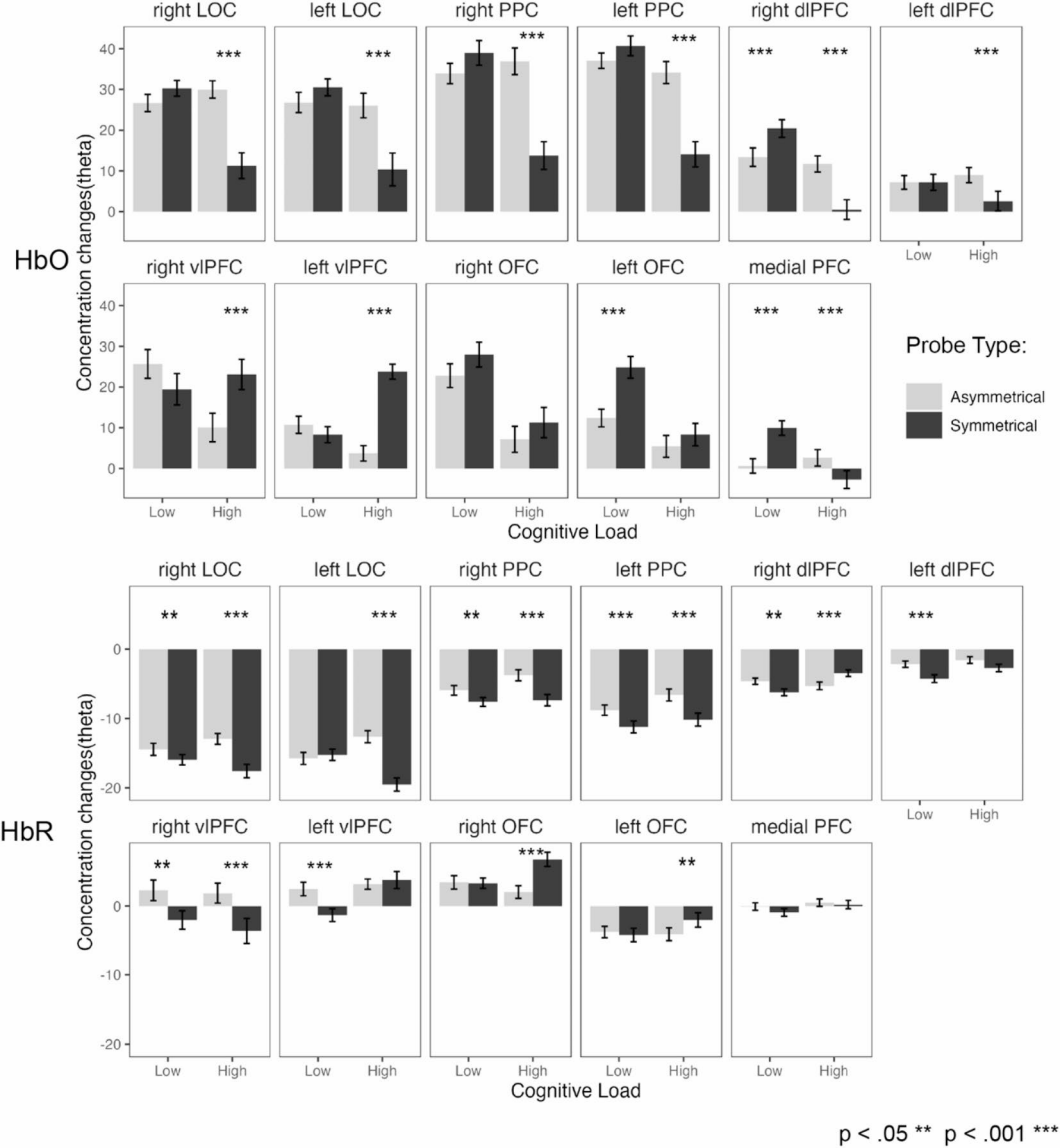
Concentration changes for HbO and HbR by ROI. Bonferroni corrected pairwise comparison between probe type and cognitive load.

The post-hoc Tukey’s HSD analysis revealed varying responses across different regions of the PFC (see Fig. 6; detailed pairwise-comparison statistics are in the Supplementary Material).

In the right dlPFC, HbO levels decreased under low cognitive load and increased under high load with symmetrical probes, while HbR levels showed the opposite pattern. The left dlPFC showed no significant change in HbO under low load but increased under high load; HbR increased under low load and trended towards significance under high load. The left OFC exhibited a significant decrease in HbO under low load, with no change under high load; HbR remained unchanged under low load but significantly decreased under high load. The right OFC showed no significant changes in HbO levels, though HbR decreased significantly under high load. In the mPFC, HbO decreased under low load and increased under high load, with no significant changes in HbR. The left vlPFC showed no significant difference in HbO under low load but a significant decrease under high load; HbR increased significantly under low load with no notable change under high load. In the right vlPFC, there was a non-significant increase in HbO under low load and a significant decrease under high load, with significant increases in HbR in both conditions. Overall, these findings indicate distinct responses to cognitive load across PFC regions, with pronounced effects in the right dlPFC, left OFC, and left vlPFC.

#### Posterior parietal cortex

In LME model analysis for HbO and HbR in right PPC, the results showed a significant effect of symmetrical probe type (*β*_*HbO*_ = 5.05, *SE*_*HbO*_ = 2.28, *p*_*HbO*_ < 0.05; *β*_*HbR*_ = -1.68, *SE*_*HbR*_ = 0.56, *p*_*HbR*_ < 0.05). Intriguingly, both measured hemoglobins significantly decreased in the interaction between probe type and cognitive load (*β*_*HbO*_ = -28.22, *SE*_*HbO*_ = 3.22, *p*_*HbO*_ < 0.001; *β*_*HbR*_ = -1.92, *SE*_*HbR*_ = 0.79, *p*_*HbR*_ < 0.001). The random effects structure indicated substantial variability across subjects, with ICC values of 0.72 for HbO and 0.67 for HbR. The model explained a significant portion of the variance in both HbO and HbR outcomes when accounting for participant variance, with conditional R^2^ values of 0.733 and 0.716, respectively.

The results in left PPC showed no consistent effect of experimental conditions on HbO and HbR changes. While we saw the effect of symmetrical probe type on HbR (*β*_*HbR*_ = -2.42, *SE*_*HbR*_ = 0.64, *p*_*HbR*_ < 0.001) the same effect was not observed in HbO (*β*_*HbO*_ = 3.65, *SE*_*HbO*_ = 2.26, *p*_*HbO*_ = 0.107). The same concerns the effect of the load (*β*_*HbO*_ = -2.82, *SE*_*HbO*_ = 2.26, *p*_*HbO*_ = 0.202; *β*_*HbR*_ = 2.18, *SE*_*HbR*_ = 0.64, *p*_*HbR*_ = 0.001) while the situation is reversed in the interaction between probe type and cognitive load (*β*_*HbO*_ = -23.73, *SE*_*HbO*_ = 3.20, *p*_*HbO*_ < 0.001; *β*_*HbR*_ = -1.13, *SE*_*HbR*_ = 0.91, *p*_*HbR*_ = 0.214). Here, as well the random effects structure in the models for HbO and HbR demonstrated significant variability among subjects, as reflected in ICC values of 0.61 and 0.71, respectively. Additionally, when considering the variance due to participants, the models accounted for a substantial part of the variance in both HbO and HbR outcomes, with conditional R^2^ values being 0.628 for HbO and 0.721 for HbR.

In a post-hoc procedure (Tukey’s HSD; see Fig. 6), the left PPC showed no significant change in HbO levels under low cognitive load between asymmetrical and symmetrical probes, but a significant increase was observed under high load. HbR concentration increased under both low and high cognitive load conditions. Similarly, the right PPC displayed no significant difference in HbO levels under low load but showed a significant increase under high load. HbR concentration also significantly increased under both low and high cognitive load conditions. These findings highlight the differentiated response of the left and right PPC to cognitive load, particularly in terms of HbO and HbR concentrations under varying cognitive demands.

#### Lateral occipital cortex

In the left LOC, an LME model revealed that the effect of probe type was not significant (*β*_*HbO*_ = 3.72, *SE*_*HbO*_ = 2.12, *p*_*HbO*_ = 0.079; *β*_*HbR*_ = 0.52, *SE*_*HbR*_ = 0.51, *p*_*HbR*_ = 0.308), nor was the effect of cognitive load (*β*_*HbO*_ = -0.74, *SE*_*Hb*_ = 2.12, *p*_*HbO*_ = 0.726). We found a statistically significant effect of accuracy differences between symmetry and asymmetry in HbO (*β*_*HbO*_ = -381.37, *SE*_*HbO*_ = 182.39, *p*_*HbO*_ < 0.05) as well as the interaction between probe type and cognitive load showed a significant decrease in HbO concentration (*β*_*HbO*_ = -19.43, *SE*_*HbR*_ = 3.00, *p*_*HbO*_ < 0.001) and HbR (*β*_*HbR*_ = -7.42, *SE*_*HbR*_ = 0.72, *p*_*HbR*_ < 0.001).

The random effects analysis indicated considerable variability at the subject level (*O*^2^ = 700.04 for HbO and *σ*^2^ = 40.70 for HbR), with the ICC suggesting that 72% of the variability in HbO and 83% of the variability in HbR could be attributed to differences between subjects. The marginal R^2^ / conditional R^2^ for HbO was 0.133 / 0.754 and for HbR was 0.031 / 0.832, indicating the random intercepts account for a relatively large proportion of the variance in the outcome.

In the analysis of the right LOC, the LME model demonstrated a significant beta weight for probe type (*β*_*HbO*_ = 3.58, *SE*_*HbO*_ = 1.77, *p*_*HbO*_ = 0.043). The effect of cognitive load approached significance (*β*_*HbO*_ = 3.29, *SE*_*HbO*_ = 1.77, *p*_*HbO*_ = 0.062). A significant interaction effect was observed between probe type and cognitive load with (*β*_*HbO*_ = -22.28, *SE*_*HbO*_ = 2.50, *p*_*HbO*_ < 0.001), suggesting a considerable decrease in HbO for the combined conditions of symmetrical probe type and high cognitive load. For HbR concentrations, there was a significant effect of probe type, (*β*_*HbR*_ = -1.51, *SE*_*HbR*_ = 0.47, *p*_*HbR*_ = 0.001), and cognitive load was significant, (*β*_*HbR*_ = 1.49, *SE*_*HbR*_ = 0.47, *p*_*HbR*_ = 0.002). The interaction between probe type and cognitive load yielded a significant effect (*β*_*HbR*_ = -3.12, *SE*_*HbR*_ = 0.67, *p*_*HbR*_ < 0.001). Random effects modeling showed substantial inter-subject variability (*σ*^2^ = 486.13 for HbO and *σ*^2^ = 34.89 for HbR), with ICC indicating that 73% of the variability in HbO and 84% of the variability in HbR could be accounted for by between-subject differences. The marginal R^2^ / conditional R^2^ for HbO was reported as 0.073 / 0.735, and for HbR as 0.038 / 0.845, indicating that the random effects accounted for a substantial proportion of the variance in the dependent variables.

The post-hoc pairwise comparison revealed significant differences in left LOC between symmetry and asymmetry for high, but not low, cognitive conditions (Table: Comparisons; see Fig. 6; both HbO and HbR). For right LOC, the pattern was similar with the exception that, in HBR, a significant difference was detected between condition also in low cognitive load. The direction of concentration changes across hemoglobins suggests the measure to be more consistent under high cognitive load in which the probe results in higher HbO concentration and lower HbR.

### Differences in cortical activations between symmetry and asymmetry (contrast)

Apart from differences in ROIs, we have tested our hypothesis about differences in the processing of symmetry and asymmetry with a channel-wise, between-conditions contrast.

**Fig. 7:**
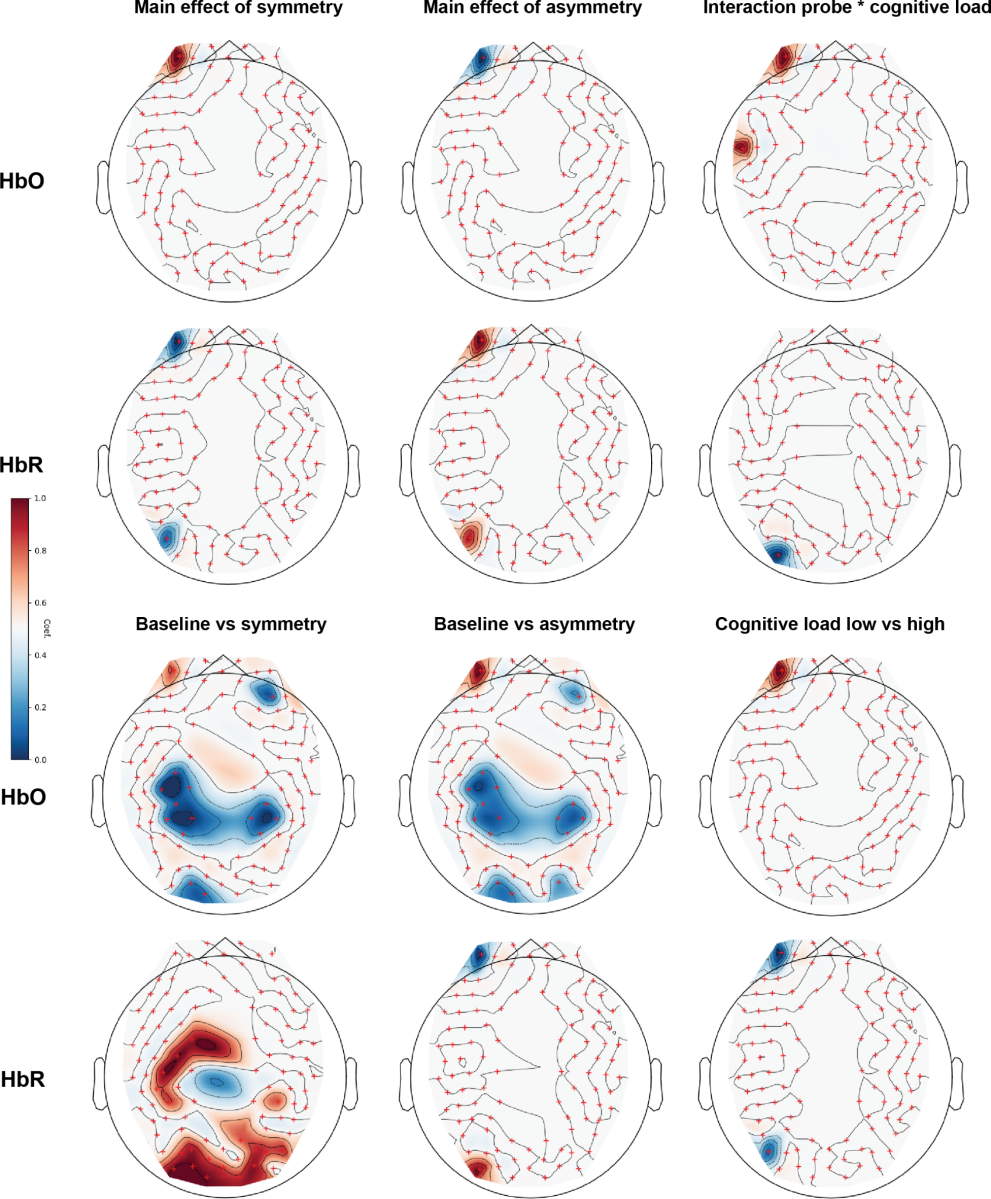
Group-level contrast map of estimated significant changes in hemoglobin concentration. All results were thresholded to *p* < 0.05 after FDR correction for multiple comparisons. Red markers denote position of the fNIRS channels. Red hues signify the increase in hemoglobin concentration, blue hues – a decrease in hemoglobin concentration.

Detailed results with anatomical registration of the channels are available in Supplementary Material: Table 8. In the group-level analysis examining the main effects of symmetry, significant effects were found in two specific channels (Fig. 7). For HbO concentration, a significant effect was observed in the left OFC (*z* = 4.63, *p* < 0.001).

For HbR concentration, the same channel demonstrated a significant decrease (Fig. 7)(*z* = -6.13, *p* < 0.001). Additionally, in the left LOC, there was a significant decrease in response to symmetry (*z* = -5.11, *p* < 0.001).

In a comparison between baseline and symmetry conditions using fNIRS, significant changes in both HbO and HbR concentrations were observed across various brain regions (Fig. 7, Supplementary Material: Table 8). For HbO, a significant increase was found in the left OFC, while notable decreases were seen in the right dlPFC and right PPC. In contrast, HbR were increased from baseline in the motor left area, right PPC, right LOC, early visual area, and left LOC, indicating a differentiated hemodynamic response to symmetry in these regions.

In examining the main effect of asymmetry on hemodynamic responses, we found a notable decrease in HbO concentration in the left OFC (*z* = -4.63, *p* < 0.001). Conversely, for HbR, significant increases were observed in the same region (z = 6.13, *p* < 0.001), and in the left LOC (*z* = 5.11, *p* < 0.001).

In the contrast between baseline and asymmetry conditions, we found notable effect in the left OFC. Here, a decrease in HbO concentration and a significant increase in HbR concentration, indicating a strong hemodynamic response to asymmetrical stimuli. Additionally, significant decreases in HbO concentration were reported in the right dlPFC, right PPC, and the early visual area of the occipital lobe, alongside increases in HbR concentration in the left LOC.

When examining the interaction between probe type and cognitive load, we found a significant interaction effect in the left OFC, reflected by an increase in HbO concentration. A similar increase in HbO was found at the junction between inferior frontal gyrus (part of vlPFC) and anterior superior temporal gyrus (aSTG). Conversely, a significant decrease in HbR concentration was noted in the left LOC.

Lastly, we investigated contrast between low and high cognitive load. Specifically, there were substantial increases in HbO concentration observed in the left OFC (*z* = 4.72, *p* < 0.001), and in the left LOC (z = 6.76, *p* < 0.001). Conversely, HbR showed significant reductions, indicating decreased oxygenation levels, in the same regions: left OFC (z = -4.61, *p* < 0.001) and left LOC (z = -5.62, *p* < 0.001).

## Discussion

Our study examined the neurocognitive and behavioral processes involved in handling symmetry and asymmetry within working memory, aiming to provide new insights into how symmetry affects complex cognitive processes. Grounded in established theories, our hypotheses tested differential cognitive mechanisms underlying these processes and how they are mirrored in cortical activity.

### Our findings

Behavioral results demonstrated that the presence of symmetry has a significant effect on task performance. Firstly, the accuracy was notably higher for symmetrical stimuli than for asymmetrical stimuli. This difference was more pronounced under high cognitive load while, under low cognitive load, the accuracy difference was minimal. Furthermore, the type of symmetry (vertical, horizontal, diagonal) also significantly affected recall accuracy, with diagonal symmetry yielding the highest accuracy under high cognitive load. Secondly, reaction time analysis revealed that symmetrical stimuli were associated with faster recall, with difference increasing under high cognitive load. We also examined error patterns, finding that symmetrical stimuli resulted in errors that were closer to the target, particularly under high cognitive load. When considering the possible role of visual complexity (i.e., the amount of information), we found that asymmetrical, more complex stimuli were associated with slightly lower accuracy and marginally slower reaction times. However, the effects measured via the current methods were minimal and unlikely to account for the observed benefits of symmetry on task performance.

In our neuroimaging investigation, we found that symmetrical probes significantly affected hemoglobin activity associated with neural response in PFC. However, the effect varied with hemoglobin and cognitive load, and was distinct across different PFC regions. For example, the right dlPFC showed notable activity in response to symmetrical probes, particularly under low cognitive load while in high load symmetry seems to result in a significant decrease in activity. The medial PFC also showed such pattern of response but with less impact on HbR. The vlPFC exhibited complex interactions. While in low cognitive load asymmetry response in HbO insignificantly overtook the symmetry response in high cognitive load symmetry response was significantly stronger than asymmetry. That said it needs to be noted that in HbR in right vlPFC in both cognitive loads the asymmetry response was higher than symmetry one. Moreover, left OFC exhibited a significant increase in HbO in response to symmetry under low cognitive load, while in HbR the effect was significant for high load condition. It is also interesting that HbO in left OFC was found to be affected by behavioral performance in the task (difference between symmetry and asymmetry). The PPC showed a significant influence of symmetrical probes, with significantly less activity in response to combined symmetry and high cognitive load. In the LOC, the effects of probe type and cognitive load were more pronounced with symmetry evoking a lower response than asymmetry under high cognitive load but the load, but was reversed in the low cognitive load condition. It was also sensitive to behavioral performance. However, only certain interactions show significant changes.

The channel-wise contrast of the effect of experimental conditions consistently demonstrates that probe type and cognitive load significantly affect the hemodynamic responses, with the magnitude of response differing between symmetry and asymmetry within the left OFC and LOC. The interaction of symmetry and cognitive load was also detected in the left hemisphere, around the junction between anterior superior temporal gyrus (aSTG) and inferior frontal gyrus (IFG). In overall, the results revealed interesting complex, region-specific patterns of brain activation in response to symmetry in visual stimuli. These patterns, although difficult to interpret, underline the intricate nature of cognitive processing of symmetry in memory tasks.

### Neurocognitive implications of symmetry

In general, the enhanced recall performance for symmetrical stimuli aligns with previous research indicating that symmetry promotes binding of visual features and aids in the organization of information within WM^33^. Faster reaction times and more accurate error patterns for symmetrical stimuli under high cognitive load further suggests symmetry supports memory via inhibitory processes, improving focus of attention on task-relevant information. Observed improvement in recall accuracy and faster reaction times for symmetrical stimuli, especially under high cognitive load, indicates that symmetry likely aids in the executive management of information. That seems to add detail to the theorized automatic effect on working memory by Rossi-Arnaud & Baddeley^6,33^. Moreover, the outcomes of our experiment suggest that the effect of symmetry on the processing in WM is superadditive (sum of responses exceeds individual effects of the experimental condition). Specifically, the enhanced recall accuracy for symmetrical stimuli, especially under high cognitive load conditions, indicates that symmetry may facilitate more efficient cognitive and retrieval processes in WM. We also investigated a potential covariance stemming from information complexity of the probe type. Our findings suggest that the advantage of the symmetry is weakly but significantly influenced by lower visual complexity. However, while more complex asymmetrical stimuli were associated with slightly lower accuracy and slower reaction times, these effects were minimal and for now insufficient to explain any substantial performance benefits observed for symmetrical stimuli. Nevertheless, overall results indicate that symmetry appears to facilitate more efficient encoding and retrieval processes, particularly under high cognitive load, where working memory resources are strained. This supports the view that symmetry offers unique cognitive advantages beyond visual simplicity.

Our neuroimaging findings reveal a complex pattern of hemodynamic responses to symmetry in task. While we observed a generally lower response to symmetry in the dlPFC and PPC under memory-intensive conditions, these patterns were not consistent across all types of hemoglobin. This suggests that while symmetry might be encoded more efficiently into working memory, as our hypothesis proposed, the relationship is nuanced and varies across different conditions. Moreover, we found no relationship in dlPFC and PPC with behavioral performance in the task, making an questionable.

Intriguingly, the response to symmetry in the ventrolateral PFC (vlPFC) and orbitofrontal cortex (OFC) contrasted with other areas. Ventrolateral PFC, is known to be implicated in cognitive and motor inhibitory control^50^. Higher activation in vlPFC may imply symmetry maybe more important to inhibitory processes under memory strain. This result is consistent across the hemoglobins in the right but not left hemisphere, supporting the notion^92^ of control processes engaged to stop or override motor responses in the task, therefore increasing chances a response. However, other results are inconsistent across the hemoglobin types and experimental conditions. Intriguingly, we also found the interaction between cognitive load and symmetry localized on the left side in the vicinity of this area.

The left OFC was the only region in the PFC where we observed a significant relationship between brain activity and behavioral differences in symmetry accuracy. The effects were predominantly seen in HbO activity, suggesting the OFC’s involvement during the task in adaptive decision-making and goal-directed behavior^93^, as well as in perceptual processing with affective elements^94^.

The lateral occipital complex (LOC) and posterior parietal cortex (PPC) also showed significant but contrasting responses to symmetry, with a notable difference in hemoglobin responses under varying cognitive loads. This reflects the complexity of how visual information quality is processed in these areas.

In the introductory section of this study, we suggested that symmetry processing within WM might engage distinct neurocognitive mechanisms, as suggested by existing theoretical frameworks. We explored the brain processes that underlie the processing and storage of symmetry during a delayed matching to sample task. Our findings in this domain provide insight into the cognitive mechanisms guiding this relationship underscoring the role of symmetry within the memory processes and cognitive control. This notion is only partially explained by the cortical activity within the brain. The intricate patterns of cortical activation observed in our study highlight the complex role of symmetry in cognitive processing and working memory. While some of our findings align with established theories, such as those by Rossi-Arnaud & Baddeley^33^, others present new insights and raise questions about the intricate mechanisms at play. These results highlight the need for further research to unravel the full impact of symmetry—both in terms of the quality and quantity of information it carries—on neurocognitive functions and their underlying neural correlates.

### Limitations

Despite the extensive significant results, it needs to be noted that the effect of differences between symmetry and asymmetry was significantly smaller than that of cognitive load. Our neuroimaging results, across all ROIs, varied in pattern of consistency. There was considerable variability in response among subjects, as evidenced by the intraclass correlation coefficients (ICC) and random effects structures. This suggests individual differences in how the brain processes symmetry and cognitive load.

Although we observed certain patterns in responses within HbO they did not reflect in HbR concentrations, raising our cautious approach in interpretation of how we identify the cortical activation with our data. In general, fNIRS hemodynamic response neural activation is indicated by an increase in HbO, and decrease in HbR. HbO is noted for being more sensitive to detect task-evoked changes more correlated with fMRI BOLD response^44,45^, however, as noted by Kinder et al.,^95^ on HbO results may lead to overinterpretation of the results. Moreover, although fNIRS offers very good temporal accuracy, its coarse spatial resolution offers us only a broad idea of the epicenters of differential activity between symmetry and asymmetry. Despite this, we see a localized effect of symmetry in PFC that offers a glimpse into mechanisms underlying symmetry processing within cognition.

## Conclusions

Our study confirmed that symmetry appears to act as a cognitive anchor, enabling better organization, retrieval and control of information in WM, thus demonstrating a compounded positive effect on memory capacity and efficiency of cognitive processes. The experiment offered insights into the neurocognitive dynamics of symmetry and asymmetry processing within WM. The observed behavioral results demonstrate that symmetry significantly enhances task performance, particularly under high cognitive loads. Symmetrical stimuli elicited distinct patterns of brain activation, which despite being difficult to interpret, highlighting the nuanced role of symmetry in cognitive processing. These findings extend our understanding of the complex interplay between visual perception and cognitive functions, underscoring symmetry’s influence on cognitive control and working memory performance. Our research not only corroborates existing theories on symmetry’s facilitative role in cognitive processes, but also opens avenues for further exploration into the intricate mechanisms of symmetry’s significance to creative cognition, environmental perception, and aesthetic valence. Thus, our study warrants further investigation of the phenomenon. Future research could build on these findings to explore the implications of symmetry processing in broader cognitive and creative contexts, enhancing our comprehension of the fundamental principles governing environmental experience.

## Electronic supplementary material

Below is the link to the electronic supplementary material.

Supplementary Material 1

Supplementary Material 2

Supplementary Material 3

Supplementary Material 4

## Data Availability

The datasets generated during this study, including both functional Near-Infrared Spectroscopy (fNIRS) and behavioral data, will be made available in the Open Science Framework (OSF) repository (https://doi.org/10.17605/OSF.IO/Z7KW3). This availability ensures that interested researchers can access the full dataset for verification and further study.

## Code availability

Alongside the data, the code used in this study, covering the fNIRS optode array design, experimental scripts, and statistical analysis from the Methods section, will also be provided in the repository. This facilitates the reproducibility of our analysis and supports further research in the field.
