# Supplementary material for: Neurocognitive dynamics and behavioral differences of symmetry and asymmetry processing in working memory: insights from fNIRS
Source: Sci Rep. 2025 Feb 8;15:4740. doi: 10.1038/s41598-024-84988-8 (PMC11807122; doi:10.1038/s41598-024-84988-8)
Supplement: Supplementary file 4 — Supplementary Material 4 [file 41598_2024_84988_MOESM4_ESM.pdf]

Supplementary Materials: Tables.

Table 1: *ANOVA Results: Accuracy ~ Probe Type * Cognitive load.*

Predictor	df_{Num}	df_{Den}	SS_{Num}	SS_{Den}	F	p	η^2_g
(Intercept)	1	36	356317.71	10597.74	1210.39	<.001	.96
Probe type	1	36	1858.16	1057.01	63.29	<.001	.11
Cognitive load	1	36	40527.29	1726.29	845.16	<.001	.73
Probe x Load	1	36	583.38	1310.91	16.02	<.001	.04

Note. df_{Num} = degrees of freedom numerator. df_{Den} = degrees of freedom denominator. SS_{Num} = sum of squares numerator. SS_{Den} = sum of squares denominator. η^2_g = generalized eta-squared.

Table 2: Summary statistics for symmetry levels by cognitive load.

Symmetry	Cognitive Load	*M*	*SD*
Diagonal	High	45.42	13.97
Diagonal	Low	67.52	13.79
Horizontal	High	35.38	12.64
Horizontal	Low	66.81	12.51
Vertical	High	33.38	10.86
Vertical	Low	67.17	13.97

Table 3: *ANOVA Results: Accuracy ~ Symmetry level * Cognitive load*

Predictor	df_{Num}	df_{Den}	*Epsilon*	SS_{Num}	SS_{Den}	*F*	*p*	η^2_g
(Intercept)	1.00	36.00		765118.93	22855.53	1205.15	<.001	.95
Cognitive load	1.00	36.00		71552.97	3114.48	827.07	<.001	.62
Symmetry	2.50	90.06	0.83	4465.22	9039.68	17.78	<.001	.09
Symmetry x Load	2.80	100.85	0.93	2295.91	8783.55	9.41	<.001	.05

Note. df_{Num} = degrees of freedom numerator. df_{Den} = degrees of freedom denominator. Epsilon = Greenhouse-Geisser multiplier for degrees of freedom, *p*-values and degrees of freedom in the table incorporate this correction. SS_{Num} = sum of squares numerator. SS_{Den} = sum of squares denominator. η^2_g = generalized eta-squared.

Table 4: *ANOVA Results: Reaction Times (correct) ~ Probe type * Cognitive load*

Predictor	df_{Num}	df_{Den}	SS_{Num}	SS_{Den}	*F*	*p*	η^2_g
(Intercept)	1	36	242991427.67	10364502.05	844.00	<.001	.95
Probe type	1	36	389902.45	245795.54	57.11	<.001	.03
Cognitive load	1	36	557305.76	757802.45	26.48	<.001	.05
Probe x Load	1	36	29865.19	208640.09	5.15	.029	.00

Table 5: *ANOVA Results: Distance of Error ~ Probe type * Cognitive load*

Predictor	df_{Num}	df_{Den}	SS_{Num}	SS_{Den}	*F*	*p*	η^2_g
(Intercept)	1	36	6135571.69	100039.67	2207.93	<.001	.97
Probe type	1	36	10456.22	30150.98	12.48	.001	.05
Cognitive load	1	36	205461.83	34739.14	212.92	<.001	.52
Probe x Load	1	36	3432.36	23508.41	5.26	.028	.02

Table 6: *ANOVA Results: Angle of error ~ Probe type * Cognitive load*

Predictor	df_{Num}	df_{Den}	SS_{Num}	SS_{Den}	F	p	η^2_g
(Intercept)	1	36	1806509.42	15358.66	4234.38	<.001	.98
Probe type	1	36	2030.50	5180.62	14.11	.001	.06
Cognitive load	1	36	33674.95	5796.79	209.13	<.001	.52
Probe x Load	1	36	463.86	4377.25	3.81	.059	.01

Table 7: *ANOVA Results: False positive rate ~ Probe type * Cognitive load*

Predictor	df_{Num}	df_{Den}	SS_{Num}	SS_{Den}	F	p	η^2_g
(Intercept)	1	36	3.53	1.40	90.76	<.001	.64
Probe type	1	36	0.00	0.13	0.49	.488	.00
Cognitive load	1	36	0.00	0.18	0.01	.916	.00
Probe x Load	1	36	0.00	0.25	0.60	.444	.00

Table 8: Differences in cortical activations (contrast). Statistical outcome by contrast and channel, with ROI identification.

contrast	Coef.	*SE*	*z*	*p*>|*z*|	*CI*[0.025	0.975]	Channel name*	ROI**
Main effect of symmetry	42.37	9.14	4.634	0.000	24.45	60.29	S2_D1 hbo	Left OFC
Main effect of symmetry	-30.00	4.89	-6.133	0.000	-39.59	-20.41	S2_D1 hbr	Left OFC
Main effect of symmetry	-25.02	4.89	-5.114	0.000	-34.61	-15.43	S32_D14 hbr	Left LOC
Main effect of asymmetry	-42.37	9.14	-4.634	0.000	-60.29	-24.45	S2_D1 hbo	Left OFC
Main effect of asymmetry	30.00	4.89	6.133	0.000	20.41	39.59	S2_D1 hbr	Left OFC
Main effect of asymmetry	25.02	4.89	5.114	0.000	15.43	34.61	S32_D14 hbr	Left LOC
Probe type x cognitive load	70.11	18.82	3.725	0.020	33.22	107.00	S2_D1 hbo	Left OFC
Probe type x cognitive load	66.51	18.82	3.534	0.020	29.62	103.40	S25_D6 hbo	Left aSTG/vlPFC
Probe type x cognitive load	-45.80	7.65	-5.986	0.000	-60.79	-30.80	S32_D15 hbr	Left LOC
Main effect of cognitive load	46.42	9.84	4.718	0.000	27.14	65.71	S2_D1 hbo	Left OFC
Main effect of cognitive load	66.50	9.84	6.759	0.000	47.22	85.78	S32_D15 hbo	Left LOC
Main effect of cognitive load	-30.08	6.52	-4.612	0.000	-42.87	-17.30	S2_D1 hbr	Left OFC
Main effect of cognitive load	-36.66	6.52	-5.620	0.000	-49.44	-23.88	S32_D15 hbr	Left LOC
Baseline vs symmetry	81.30	28.33	2.870	0.032	25.78	136.83	S2_D1 hbo	Left OFC
Baseline vs symmetry	-105.13	28.33	-3.711	0.004	-160.66	-49.61	S9_D19 hbo	Right dlPFC
Baseline vs symmetry	-84.63	28.33	-2.987	0.024	-140.15	-29.10	S17_D24 hbo	Right PPC
Baseline vs symmetry	-89.21	28.33	-3.149	0.018	-144.73	-33.69	S17_D26 hbo	Right PPC
Baseline vs symmetry	-104.78	28.33	-3.699	0.004	-160.30	-49.25	S19_D24 hbo	Right PPC
Baseline vs symmetry	-85.54	28.33	-3.020	0.024	-141.07	-30.02	S19_D26 hbo	Right PPC
Baseline vs symmetry	-89.88	28.33	-3.173	0.018	-145.40	-34.35	S23_D15 hbo	Early Visual
Baseline vs symmetry	-105.65	28.33	-3.729	0.004	-161.17	-50.13	S26_D7 hbo	Motor Left
Baseline vs symmetry	-125.75	28.33	-4.439	0.001	-181.27	-70.22	S26_D9 hbo	Motor Left
Baseline vs symmetry	-80.60	28.33	-2.845	0.032	-136.12	-25.08	S28_D7 hbo	Motor Left
Baseline vs symmetry	-106.53	28.33	-3.760	0.004	-162.05	-51.00	S28_D9 hbo	Left PPC
Baseline vs symmetry	-122.25	28.33	-4.315	0.001	-177.77	-66.72	S28_D11 hbo	Left PPC

Baseline vs symmetry	-99.25	28.33	-3.503	0.007	-154.77	-43.72	S30_D9 hbo	Left PPC
Baseline vs symmetry	-84.33	28.33	-2.977	0.024	-139.85	-28.81	S30_D11 hbo	Left PPC
Baseline vs symmetry	-78.79	28.33	-2.781	0.036	-134.31	-23.27	S31_D15 hbo	Left LOC
Baseline vs symmetry	52.98	12.83	4.131	0.001	27.84	78.12	S8_D7 hbr	Motor Left
Baseline vs symmetry	38.54	12.83	3.005	0.018	13.40	63.68	S19_D24 hbr	Right PPC
Baseline vs symmetry	37.14	12.83	2.896	0.024	12.00	62.28	S21_D28 hbr	Right LOC
Baseline vs symmetry	45.51	12.83	3.548	0.004	20.37	70.65	S22_D28 hbr	Right LOC
Baseline vs symmetry	44.13	12.83	3.441	0.005	18.99	69.27	S22_D29 hbr	Right LOC
Baseline vs symmetry	56.18	12.83	4.380	0.000	31.04	81.32	S22_D30 hbr	Right LOC
Baseline vs symmetry	58.43	12.83	4.555	0.000	33.29	83.56	S23_D15 hbr	Early Visual
Baseline vs symmetry	36.81	12.83	2.870	0.024	11.67	61.95	S23_D16 hbr	Early Visual
Baseline vs symmetry	43.52	12.83	3.393	0.006	18.38	68.66	S23_D30 hbr	Early Visual
Baseline vs symmetry	46.07	12.83	3.592	0.004	20.93	71.21	S24_D29 hbr	Right LOC
Baseline vs symmetry	59.34	12.83	4.627	0.000	34.21	84.48	S26_D7 hbr	Motor Left
Baseline vs symmetry	60.79	12.83	4.739	0.000	35.65	85.93	S26_D9 hbr	Motor Left
Baseline vs symmetry	34.90	12.83	2.721	0.036	9.76	60.04	S27_D9 hbr	Left PPC
Baseline vs symmetry	42.38	12.83	3.304	0.007	17.24	67.52	S30_D9 hbr	Left PPC
Baseline vs symmetry	51.98	12.83	4.052	0.001	26.84	77.12	S31_D13 hbr	Left LOC
Baseline vs symmetry	39.66	12.83	3.092	0.014	14.52	64.80	S31_D14 hbr	Left LOC
Baseline vs symmetry	60.22	12.83	4.695	0.000	35.08	85.36	S31_D15 hbr	Left LOC
Baseline vs symmetry	34.48	12.83	2.688	0.038	9.34	59.62	S31_D16 hbr	Early Visual
Baseline vs symmetry	60.43	12.83	4.712	0.000	35.29	85.57	S32_D15 hbr	Left LOC
Baseline vs asymmetry	166.04	33.16	5.007	0.000	101.05	231.03	S2_D1 hbo	Left OFC
Baseline vs asymmetry	-99.30	33.16	-2.995	0.021	-164.29	-34.31	S9_D19 hbo	Right dlPFC
Baseline vs asymmetry	-93.49	33.16	-2.819	0.032	-158.48	-28.50	S17_D24 hbo	Right PPC
Baseline vs asymmetry	-101.41	33.16	-3.058	0.020	-166.40	-36.42	S17_D26 hbo	Right PPC
Baseline vs asymmetry	-119.58	33.16	-3.606	0.008	-184.57	-54.59	S19_D24 hbo	Right PPC
Baseline vs asymmetry	-102.12	33.16	-3.080	0.020	-167.11	-37.13	S19_D26 hbo	Right PPC

baseline vs asymmetry	-90.60	33.16	-2.732	0.039	-155.59	-25.61	S22_D30 hbo	Right LOC
baseline vs asymmetry	-100.11	33.16	-3.019	0.021	-165.10	-35.11	S23_D15 hbo	Early Visual
baseline vs asymmetry	-111.74	33.16	-3.370	0.013	-176.73	-46.75	S26_D7 hbo	Motor Left
baseline vs asymmetry	-127.94	33.16	-3.858	0.004	-192.93	-62.95	S26_D9 hbo	Motor Left
baseline vs asymmetry	-104.99	33.16	-3.166	0.019	-169.98	-40.00	S28_D7 hbo	Motor Left
baseline vs asymmetry	-117.84	33.16	-3.554	0.008	-182.83	-52.85	S28_D9 hbo	Left PPC
baseline vs asymmetry	-134.82	33.16	-4.066	0.002	-199.81	-69.83	S28_D11 hbo	Left PPC
baseline vs asymmetry	-107.35	33.16	-3.238	0.017	-172.35	-42.36	S30_D9 hbo	Left PPC
baseline vs asymmetry	-102.68	33.16	-3.097	0.020	-167.67	-37.69	S30_D11 hbo	Left PPC
baseline vs asymmetry	-97.33	33.16	-2.935	0.024	-162.32	-32.34	S31_D15 hbo	Left LOC
baseline vs asymmetry	-79.84	18.65	-4.281	0.002	-116.40	-43.29	S2_D1 hbr	Left OFC
baseline vs asymmetry	74.75	18.65	4.008	0.003	38.20	111.31	S32_D15 hbr	Left LOC

*Hemoglobin type: hbo (oxygenated), hbr (deoxygenated)

** Anatomical registration of channels available in Supplementary Material: ROI and Optode position.

Supplementary Materials: Behavioral Results. Extended Tables.

Table 9: Pairwise comparisons of accuracy in symmetry types and cognitive load. (Bonferroni corrected p-value)

Comparison	$M_{difference}$	*SE*	*df*	*t*	*p*
Asymmetry Low - Diagonal Low	-4.00	2.14	238	-1.87	1
Asymmetry Low - Horizontal Low	-2.42	2.14	238	-1.13	1
Asymmetry Low - Vertical Low	-2.97	2.14	238	-1.39	1
Diagonal Low - Horizontal Low	1.59	2.14	238	0.74	1
Diagonal Low - Vertical Low	1.03	2.14	238	0.48	1
Diagonal Low - Diagonal High	23.04	2.14	238	10.76	0
Horizontal Low - Vertical Low	-0.56	2.14	238	-0.26	1
Horizontal Low - Horizontal High	30.37	2.14	238	14.18	0
Asymmetry High - Horizontal High	-9.18	2.14	238	-4.29	0.001
Asymmetry High - Vertical High	-6.44	2.14	238	-3.01	0.082
Diagonal High - Horizontal High	8.91	2.14	238	4.16	0.001
Diagonal High - Vertical High	11.66	2.14	238	5.44	0
Horizontal High - Vertical High	2.74	2.14	238	1.28	1
Vertical Low - Vertical High	33.67	2.14	238	.128	1

Supplementary Material: Neuroimaging Results. Linear Mixed Effects Model Tables.

Table 10: Left OFC

	HbO				HbR			
Predictors	*Estimates*	*SE*	*CI [2.5-97.5]*	*p*	*Estimates*	*SE*	*CI [2.5-97.5]*	*p*
(Intercept)	-25.09	37.85	-102.10 –51.93	0.508	-9.63	15.16	-40.47 – 21.20	0.525
Probe Type [Symmetrical]	12.42	1.85	8.79 – 16.04	**<0.001**	-0.43	0.65	-1.69 – 0.84	0.510
Cognitive Load [High]	-6.96	1.85	-10.58 – -3.33	**<0.001**	-0.31	0.65	-1.58 – 0.95	0.627
Symmetry-Asymmetry*	324.19	156.51	5.75 – 642.62	**0.039**	32.85	62.68	-94.68 – 160.38	0.600
Low-High Load*	55.34	109.08	-166.59 – 277.27	0.612	12.34	43.69	-76.54 – 101.22	0.778
Probe Type [Symmetrical] × Cognitive Load [High]	-9.53	2.61	-14.66 – -4.40	**<0.001**	2.48	0.91	-0.68 – 4.27	**0.007**
**Random Effects**								
σ^2	533.16				65.25			
τ_{00}	1302.03 $_{sub}$				209.26 $_{sub}$			
ICC	0.71				0.76			
N	26 $_{sub}$				26 $_{sub}$			
Observations	1248				1248			
Marginal R^2 / Conditional R^2	0.129 / 0.747				0.012 / 0.765			
**Model fit (AIC/σ):**	11517.71/23.09				8903.12/8.07			

**Behavioural accuracy difference*

Table 11: Right OFC

	HbO				HbR			
Predictors	*Estimates*	*std. Error*	*CI*	*p*	*Estimates*	*std. Error*	*CI*	*p*
(Intercept)	-21.45	49.91	-122.99 – 80.10	0.667	-10.60	13.89	-38.85 – 17.66	0.446
Probe Type [Symmetrical]	5.18	2.07	1.11 – 9.23	**0.012**	-0.13	0.71	-1.52 – 1.25	0.850
Encoding Load [High]	-15.61	2.07	-19.66 – -11.55	**<0.001**	-1.38	0.71	-2.77 – 0.007	0.051
Symmetry-Asymmetry*	378.20	206.39	-41.73 – 798.13	0.067	62.52	57.42	-54.32 – 179.35	0.277
Low-High Load*	66.17	143.85	-226.50 – 358.84	0.646	32.60	40.02	-48.83 – 114.03	0.415
Probe Type [Symmetrical] × Cognitive Load [High]	-1.07	2.92	-6.80 – 4.66	0.714	4.83	1.00	2.85 – 6.79	**<0.001**
**Random Effects**								
σ^2	667.33				78.50			
τ_{00}	2269.73 sub				175.14 sub			
ICC	0.77				0.69			
N	26 sub				26 sub			
Observations	1248				1248			
Marginal R^2 / Conditional R^2	0.111 / 0.798				0.050 / 0.706			
**Model fit (AIC/σ):**	11806.41/25.83				9124.51/8.85			

Table 12: Left vlPFC

	HbO				HbR			
Predictors	*Estimates*	*std. Error*	*CI*	*p*	*Estimates*	*std. Error*	*CI*	*p*
(Intercept)	13.58	25.20	-37.68 – 64.84	0.590	-10.16	14.89	-40.45 – 20.12	0.495
Probe Type [Symmetrical]	-2.42	1.83	-6.01 – 1.16	0.185	-3.78	0.78	-5.31 – 2.24	**<0.001**
Cognitive Load [High]	-6.99	1.83	-10.58 – -3.40	**<0.001**	0.70	0.78	-0.83 – 2.23	0.370
Symmetry-Asymmetry*	187.63	104.13	-24.22 – 399.49	0.072	30.70	61.54	-94.51 – 155.92	0.618
Low-High Load*	-47.31	72.57	-194.96 – 100.35	0.515	34.56	42.89	-52.71 – 121.83	0.421
Probe Type [Symmetrical] × Cognitive Load [High]	22.47	2.59	-17.39 – 27.53	**<0.001**	4.37	1.11	2.19 – 6.54	**<0.001**
**Random Effects**								
σ^2	521.44				95.59			
τ_{00}	570.39 $_{sub}$				201.06 $_{sub}$			
ICC	0.52				0.68			
N	26 $_{sub}$				26 $_{sub}$			
Observations	1248				1248			
Marginal R^2 / Conditional R^2	0.123 / 0.581				0.032 / 0.688			
**Model fit (AIC/σ):**	11469.37/22.83				9368.93/9.77			

Table 13: Right vlPFC

	HbO				HbR			
Predictors	*Estimates*	*std. Error*	*CI*	*p*	*Estimates*	*std. Error*	*CI*	*p*
(Intercept)	17.56	54.86	-94.04 – 129.17	0.749	3.78	23.14	-43.40 – 50.86	0.870
Probe Type [Symmetrical]	-6.20	2.67	-11.42 – -0.97	**0.020**	-4.29	1.19	-6.62 – 1.91	**<0.001**
Cognitive Load [High]	-15.60	2.67	-20.82 – -10.37	**<0.001**	-0.42	1.19	-2.76 – 1.91	0.721
Symmetry-Asymmetry*	350.19	226.81	-111.29 – 811.66	0.123	-76.66	95.68	-271.33 – 118.01	0.423
Low-High Load*	-44.88	158.08	-366.51 – 276.75	0.777	10.67	66.69	-125.0 – 146.35	0.873
Probe Type [Symmetrical] × Cognitive Load [High]	19.23	3.77	11.84 – 26.62	**<0.001**	-1.15	1.69	-4.46 – 2.15	0.494
**Random Effects**								
σ^2	1108.21				221.63			
τ_{00}	2734.77 $_{sub}$				486.17 $_{sub}$			
ICC	0.71				0.69			
N	26 $_{sub}$				26 $_{sub}$			
Observations	1248				1248			
Marginal R^2 / Conditional R^2	0.079 / 0.734				0.028 / 0.696			
**Model fit (AIC/σ):**	12431.13/33.28				10419.43/14.88			

Table 14: Medial PFC

	HbO				HbR			
Predictors	*Estimates*	*std. Error*	*CI*	*p*	*Estimates*	*std. Error*	*CI*	*p*
(Intercept)	-8.23	30.49	-70.26 – 53.79	0.787	5.79	8.41	-17.15 – 10.14	0.491
Probe Type [Symmetrical]	9.29	1.25	6.84 – 11.74	**<0.001**	-0.85	0.45	-3.00 – -1.16	0.057
Cognitive Load [High]	1.99	1.25	-0.45 – 4.44	0.111	0.56	0.45	-0.32 – 1.51	0.207
Symmetry-Asymmetry*	206.79	126.07	-49.71 – 463.29	0.101	-36.20	34.76	-106.54 – 6.29	0.298
Low-High Load*	-13.30	87.87	-192.07 – 253.45	0.880	-11.63	24.22	-24.82 – 53.82	0.631
Probe Type [Symmetrical] × Cognitive Load [High]	-14.63	1.77	-10.81 – -1.92	**<0.001**	0.57	0.63	-0.35 – 2.24	0.364
**Random Effects**								
σ^2	243.73				30.90			
τ_{00}	846.96 $_{sub}$				64.12 $_{sub}$			
ICC	0.78				0.67			
N	26 $_{sub}$				26 $_{sub}$			
Observations	1248				1248			
Marginal R^2 / Conditional R^2	0.098 / 0.798				0.032 / 0.685			
**Model fit (AIC/σ):**	10549.95/15.61				7959.09/5.55			

Table 15: Left dlPFC

	HbO				HbR			
Predictors	*Estimates*	*std. Error*	*CI*	*p*	*Estimates*	*std. Error*	*CI*	*p*
(Intercept)	-20.42	29.88	-81.20 – 40.37	0.495	-3.51	6.71	-17.15 – 10.14	0.601
Probe Type [Symmetrical]	-0.01	1.60	-3.15 – 3.13	0.996	-2.09	0.47	-3.00 – -1.16	**<0.001**
Cognitive Load [High]	1.76	1.60	-1.38 – 4.90	0.272	0.60	0.47	-0.32 – 1.51	0.204
Symmetry-Asymmetry*	53.55	123.52	-197.77 – 304.87	0.665	-50.12	27.73	-106.54 – 6.29	0.071
Low-High Load*	78.29	86.09	-98.87 – 253.45	0.363	14.50	19.33	-24.82 – 53.82	0.453
Probe Type [Symmetrical] × Cognitive Load [High]	-6.37	2.27	-10.81 – -1.92	**0.005**	0.94	0.66	-0.35 – 2.24	0.155
**Random Effects**								
σ^2	400.63				34.36			
τ_{00}	809.62 $_{sub}$				40.51 $_{sub}$			
ICC	0.67				0.54			
N	26 $_{sub}$				26 $_{sub}$			
Observations	1248				1248			
Marginal R^2 / Conditional R^2	0.027 / 0.678				0.100 / 0.587			
**Model fit (AIC/σ):**	11156.20/20.01				8077.11/5.86			

Table 16: Right dlPFC

	HbO				HbR			
Predictors	*Estimates*	*std. Error*	*CI*	*p*	*Estimates*	*std. Error*	*CI*	*p*
(Intercept)	18.00	35.67	-54.57 – 90.57	0.614	-1.80	6.70	-15.43 – 11.84	0.789
Probe Type [Symmetrical]	7.03	1.50	4.08 – 9.98	**<0.001**	-1.61	0.48	-2.55 – -0.67	**0.001**
Cognitive Load [High]	-1.68	1.50	-4.26 – 1.27	0.265	-0.67	0.48	-1.60 – 0.26	0.159
Symmetry-Asymmetry*	47.21	147.51	-252.91 – 347.33	0.749	-17.76	27.70	-74.12 – 0.26	0.522
Low-High Load*	-24.52	102.81	-233.69 – 184.65	0.812	-5.51	19.31	-44.79 – 33.77	0.775
Probe Type [Symmetrical] × Cognitive Load [High]	-18.24	2.12	-22.41 – -14.07	**<0.001**	3.45	0.67	2.12 – 4.76	**<0.001**
**Random Effects**								
σ^2	352.22				35.51			
τ_{00}	1159.12 $_{sub}$				40.41 $_{sub}$			
ICC	0.77				0.53			
N	26 $_{sub}$				26 $_{sub}$			
Observations	1248				1248			
Marginal R^2 / Conditional R^2	0.038 / 0.776				0.022 / 0.543			
**Model fit (AIC/σ):**	11008.03/18.76				8117.43/5.95			

Table 17: Left PPC

	HbO				HbR			
Predictors	*Estimates*	*std. Error*	*CI*	*p*	*Estimates*	*std. Error*	*CI*	*p*
(Intercept)	28.57	36.99	-46.67 – 103.81	0.440	-11.21	13.27	-38.21 – 15.80	0.399
Probe Type [Symmetrical]	3.65	2.26	-0.78 – 8.09	0.107	-2.42	0.64	-3.67 – -1.16	**<0.001**
Cognitive Load [High]	-2.89	2.26	-7.32 – 1.55	0.202	2.18	0.64	0.92 – 3.44	**0.001**
Symmetry-Asymmetry*	-26.56	152.88	-337.61 – 284.50	0.862	-26.30	54.88	-137.96 – 85.36	0.632
Low-High Load*	32.75	106.55	-184.04 – 249.54	0.759	13.13	38.25	-64.69 – 90.96	0.731
Probe Type [Symmetrical] × Cognitive Load [High]	-23.73	3.20	-30.00 – -17.45	**<0.001**	-1.13	0.91	-2.90 – 0.65	0.214
**Random Effects**								
σ^2	798.54				64.25			
τ_{00}	1236.36 $_{sub}$				160.14 $_{sub}$			
ICC	0.61				0.71			
N	26 $_{sub}$				26 $_{sub}$			
Observations	1248				1248			
Marginal R^2 / Conditional R^2	0.053 / 0.628				0.025 / 0.721			
**Model fit (AIC/σ):**	12010.14/28.25				8877.49/8.01			

Table 18: Right PPC

	HbO				HbR			
Predictors	*Estimates*	*std. Error*	*CI*	*p*	*Estimates*	*std. Error*	*CI*	*p*
(Intercept)	48.84	48.04	-48.88 – 146.57	0.309	-17.24	10.44	-38.47 – 4.00	0.099
Probe Type [Symmetrical]	5.05	2.28	0.58 – 9.51	**0.027**	-1.68	0.56	-2.78 – -0.58	**0.003**
Cognitive Load [High]	3.00	2.28	-1.47 – 7.46	0.189	2.17	0.56	1.07 – 3.26	**<0.001**
Symmetry-Asymmetry*	44.39	198.61	-359.71 – 448.49	0.823	-59.03	43.16	-146.84 – 28.79	0.172
Low-High Load*	-57.20	138.43	-338.84 – 224.44	0.680	48.51	30.08	-12.69 – 109.71	0.107
Probe Type [Symmetrical] × Cognitive Load [High]	-28.22	3.22	-34.54 – -21-90	**<0.001**	-1.92	0.79	-3.47 – -0.36	**0.015**
**Random Effects**								
σ^2	809.41				48.85			
τ_{00}	2097.84 $_{sub}$				98.85 $_{sub}$			
ICC	0.72				0.67			
N	26 $_{sub}$				26 $_{sub}$			
Observations	1248				1248			
Marginal R^2 / Conditional R^2	0.041 / 0.733				0.141 / 0.716			
**Model fit (AIC/σ):**	12040.28/28.45				8530.16/6.98			

Table 19: Left LOC

	HbO				HbR			
Predictors	*Estimates*	*std. Error*	*CI*	*p*	*Estimates*	*std. Error*	*CI*	*p*
(Intercept)	89.50	44.11	-0.24 – 179.24	**0.043**	-11.43	14.60	-41.13 – 18.28	0.434
Probe Type [Symmetrical]	3.72	2.12	-0.43 – 7.87	0.079	0.52	0.51	-0.48 – -1.52	0.308
Cognitive Load [High]	-0.74	2.12	-4.89 – 3.41	0.726	3.14	0.51	2.13 – 4.13	**<0.001**
Symmetry-Asymmetry*	-381.37	182.39	-752.45 – 10.27	**0.037**	-26.23	60.38	-149.08 – 96.63	0.664
Low-High Load*	-125.22	127.12	-383.85 – 133.40	0.325	-8.68	42.09	-94.31 – 76.94	0.837
Probe Type [Symmetrical] × Cognitive Load [High]	-19.43	3.00	-25.30 – -13.55	**<0.001**	-7.42	0.72	-8.83 – -5.99	**<0.001**
**Random Effects**								
σ^2	700.04				40.70			
τ_{00}	1768.68 $_{sub}$				194.63 $_{sub}$			
ICC	0.72				0.83			
N	26 $_{sub}$				26 $_{sub}$			
Observations	1248				1248			
Marginal R^2 / Conditional R^2	0.133 / 0.754				0.031 / 0.832			
**Model fit (AIC/σ):**	11858.44/26.45				8324.37/6.37			

Table 20: Right LOC

	HbO				HbR			
Predictors	*Estimates*	*std. Error*	*CI*	*p*	*Estimates*	*std. Error*	*CI*	*p*
(Intercept)	62.18	36.60	-12.27 – 136.64	0.090	-11.41	14.09	-40.07 – 17.25	0.418
Probe Type [Symmetrical]	3.58	1.77	-0.11 – 7.04	**0.043**	-1.51	0.47	-2.44 – -0.58	**0.001**
Cognitive Load [High]	3.29	1.77	-0.16 – 6.75	0.062	1.49	0.47	-0.56 – 2.42	**0.002**
Symmetry-Asymmetry*	-169.80	151.32	-477.67 – 138.07	0.262	-50.33	58.26	-168.87 – 68.20	0.388
Low-High Load*	-80.26	105.46	-294.84 – 134.31	0.447	0.42	40.61	-82.19 – 83.04	0.992
Probe Type [Symmetrical] × Cognitive Load [High]	-22.28	2.50	-27.17 – -17.38	**<0.001**	-3.12	0.67	-4.42 – -1.80	**<0.001**
**Random Effects**								
σ^2	486.13				34.89			
τ_{00}	1217.37 $_{sub}$				181.25 $_{sub}$			
ICC	0.71				0.84			
N	26 $_{sub}$				26 $_{sub}$			
Observations	1248				1248			
Marginal R^2 / Conditional R^2	0.073 / 0.735				0.038 / 0.845			
**Model fit (AIC/σ):**	11403.12/22.04				8134.38/5.90			

Table 21: Early Visual Area

	HbO				HbR			
Predictors	*Estimates*	*std. Error*	*CI*	*p*	*Estimates*	*std. Error*	*CI*	*p*
(Intercept)	81.29	46.47	-13.26 – 175.83	0.081	-14.87	13.63	-42.60 – 12.85	0.275
Probe Type [Symmetrical]	4.70	2.20	0.38 – 9.02	**0.033**	-2.08	0.50	-3.06 – -1.09	**<0.001**
Cognitive Load [High]	2.21	2.20	-2.11 – 6.52	0.317	0.81	0.50	-0.18 – 1.79	0.109
Symmetry-Asymmetry*	-431.61	192.15	-822.56 – -40.66	**0.025**	-3.09	56.36	-117.75 – 111.58	0.956
Low-High Load*	-103.24	133.92	-375.71 – 169.24	0.441	7.27	39.28	-72.65 – 87.19	0.853
Probe Type [Symmetrical] × Cognitive Load [High]	-22.22	3.11	-28.32 – -16.11	**<0.001**	-3.63	0.71	-5.03 – -2.23	**<0.001**
**Random Effects**								
σ^2	755.67				39.55			
τ_{00}	1963.56 $_{sub}$				169.46 $_{sub}$			
ICC	0.72				0.81			
N	26 $_{sub}$				26 $_{sub}$			
Observations	1248				1248			
Marginal R^2 / Conditional R^2	0.142 / 0.762				0.024 / 0.815			
**Model fit (AIC/σ):**	11954.60/27.48				8285.89/6.28			
